# Sustained expression of the transcription factor GLIS3 is required for normal beta cell function in adults

**DOI:** 10.1002/emmm.201201398

**Published:** 2012-11-29

**Authors:** Yisheng Yang, Benny Hung-Junn Chang, Lawrence Chan

**Affiliations:** 1 Division of Diabetes, Endocrinology and Metabolism, Department of Medicine, Diabetes and Endocrinology Research Center, Baylor College of MedicineHouston, TX, USA; 2 Department of Molecular and Cellular Biology, Baylor College of MedicineHouston, TX, USA; 3 Department of Internal Medicine, St. Luke's Episcopal HospitalHouston, TX, USA

**Keywords:** beta cells, cyclin D2, diabetes, *Glis3*, insulin

## Abstract

Genome-wide association studies identified *GLIS3* as a susceptibility locus for type 1 and type 2 diabetes. Global *Glis3* deficiency in mice leads to congenital diabetes and neonatal lethality. In this study, we explore the role of *Glis3* in adulthood using *Glis3*^+/−^ and conditional knockout animals. We challenged *Glis3*^+/−^ mice with high fat diet for 20 weeks and found that they developed diabetes because of impaired beta cell mass expansion. GLIS3 controls beta cell proliferation in response to high-fat feeding at least partly by regulating *Ccnd2* transcription. To determine if sustained *Glis3* expression is essential to normal beta cell function, we generated *Glis3*^*fl*/*fl*^/*Pdx1Cre*^*ERT*+^ animal by intercrossing *Glis3*^*fl*/*fl*^ mice with *Pdx1Cre*^*ERT*+^ mice and used tamoxifen (TAM) to induce *Glis3* deletion in adults. Adult *Glis3*^*fl*/*fl*^/*Pdx1Cre*^*ERT*+^ mice are euglycaemic. TAM-mediated beta cell-specific inactivation of *Glis3* in adult mice downregulates insulin expression, leading to hyperglycaemia and subsequently enhanced beta cell apoptosis. We conclude that normal *Glis3* expression is required for pancreatic beta cell function and mass maintenance during adulthood, which impairment leads to diabetes in adults.

## INTRODUCTION

*Glis3*, a member of the Krüppel-like family of transcription factors (Kim et al, 2003), is highly expressed in pancreatic beta cells (Senee et al, 2006; Yang et al, 2009). *GLIS3* mutations were found to cause sporadic neonatal diabetes in humans (Senee et al, 2006). We and others reported defective islet cell differentiation and lethal neonatal diabetes in *Glis3*-deficient mice (Kang et al, 2009; Watanabe et al, 2009; Yang et al, 2011). We further showed that GLIS3 binds to Glis3-response elements (Glis3REs) in the *Ngn3* promoter, activating *Ngn3* directly and synergistically with hepatocyte nuclear factor 6 (HNF6) and forkhead box protein A2 (FOXA2), uncovering a pivotal role of *Glis3* in beta cell function during embryogenesis (Yang et al, 2011).

In addition to its role in foetal islet development, there is evidence that *Glis3* may also be involved in the regulation of adult beta cell function. Recent genome-wide association studies (GWAS) in adult populations identified *GLIS3* as a candidate gene for type 1 diabetes (Barrett et al, 2009), and as a gene that is associated with type 2 diabetes (Cho et al, 2012; Dupuis et al, 2010; Liu et al, 2011; Rees et al, 2011). Variants at *GLIS3* were associated with beta cell dysfunction in the latter group (Boesgaard et al, 2010). Moreover, the *GLIS3* locus is linked to altered fasting glucose level in healthy children and adolescents (Barker et al, 2011). These population studies suggest that *Glis3* may regulate beta cell function during adolescence and adulthood.

*Glis3*^−/−^ mice die neonatally (Kang et al, 2009; Watanabe et al, 2009; Yang et al, 2011), which precludes the use of this model to investigate the role of *Glis3* in adult animals. In order to gain insight into the function of *Glis3* in adults, we generated two independent mouse models. First, we studied *Glis3*^+/−^ mice which were euglycaemic and grew normally. Intriguingly, we found that haploinsufficiency of *Glis3* made the adult *Glis3*^+/−^ mice much more prone than wild-type to develop high fat diet (HFD)-induced diabetes due to an impairment of beta cell proliferation and beta cell mass expansion in response to HFD. Second, we generated conditional *Glis3*-inactivated mice by crossing *Glis3*^*fl*/*fl*^ mice (Yang et al, 2011) with *Pdx1-Cre*^*ERT*+^ (Gannon et al, 2000) mice. We showed that tamoxifen (TAM)-induced deletion of *Glis3* in adult animals leads to acute downregulation of insulin production, hyperglycaemia and subsequently beta cells apoptosis and fulminant diabetes. These findings provide the molecular basis for the *Glis3* locus playing a key role in glycaemic control in the adult population.

## RESULTS

### *Glis3*^+/−^ mice develop diabetes after high-fat feeding

**Figure 1:**
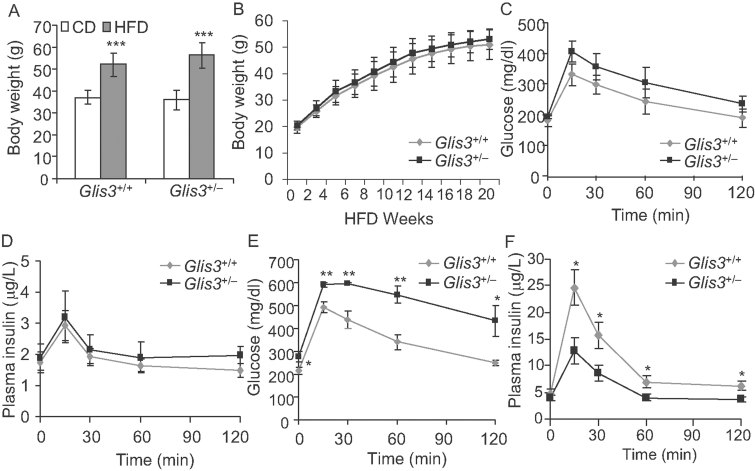
*Glis3*^+/−^ mice develop diabetes in response to HFD-feeding **A.** Body weights of *Glis3*^+/+^ and *Glis3*^+/−^ mice fed with a CD (*n* = 5) or HFD (*n* = 7) for 20 weeks. ***p = 0.0003 *versus* CD.**B.** Growth curve of *Glis3*^+/+^ (*n* = 8) and *Glis3*^+/−^ (*n* = 12) mice fed with a HFD for 20 weeks.**C,D.** After 6 h fast, gavage GTT (1.5 g/Kg BW) was performed in *Glis3*^+/+^ and *Glis3*^+/−^ mice fed with regular CD for 20 weeks. Plasma glucose (**C**) and insulin (**D**) were measured at time 0, 15, 30, 60 and 120 min after glucose injection. *n* = 5 for each group.**E,F.** Gavage GTT (2 g/kg BW) was performed in *Glis3*^+/+^ (*n* = 7) and *Glis3*^+/−^ (*n* = 5) mice fed with a HFD for 20 weeks with 6 h fast. Plasma glucose (**E**) and insulin (**F**) were measured at indicated time points. Results were analysed by student's *t*-test and presented as the mean ± SE. Gavage GTT: *p < 0.05; **p < 0.01, *versus Glis3*^+/+^. Insulin during GTT: *p = 0.028, 0.029, 0.048, 0.045 at 15, 30, 60 and 120 min after glucose injection, respectively.

The neonatal lethality in *Glis3*^−/−^ mice (Kang et al, 2009; Watanabe et al, 2009; Yang et al, 2011) prevents us from investigating the role of homozygous loss of *Glis3* in the adult pancreas in these mice. We, therefore, examined *Glis3*'s function in heterozygotes. *Glis3*^+/−^ and *Glis3*^+/+^ mice exhibited similar body weights whether they were put on a regular chow diet (CD) or a HFD for 20 weeks (Fig 1A and B). While on a CD for 20 weeks, *Glis3*^+/−^ and *Glis3*^+/+^ mice displayed similar fasting blood glucose, glucose tolerance (Fig 1C) and plasma insulin during glucose tolerance test (GTT; Fig 1D), although the *Glis3*^+/−^ showed a trend towards higher plasma glucose compared to *Glis3*^+/+^ controls after glucose challenge (Fig 1C). However, after HFD feeding for 20 weeks, *Glis3*^+/−^ mice developed diabetes with significantly higher fasting blood glucose (Fig 1E). They also displayed increased blood glucose but lower plasma insulin levels during GTT (Fig 1F), as compared to *Glis3*^+/+^ controls, indicating severe pancreatic beta cell dysfunction in adult *Glis3*^+/−^ mice in response to HFD feeding.

### *Glis3*^+/−^ mice fail to expand beta cell mass in response to HFD feeding

**Figure 2:**
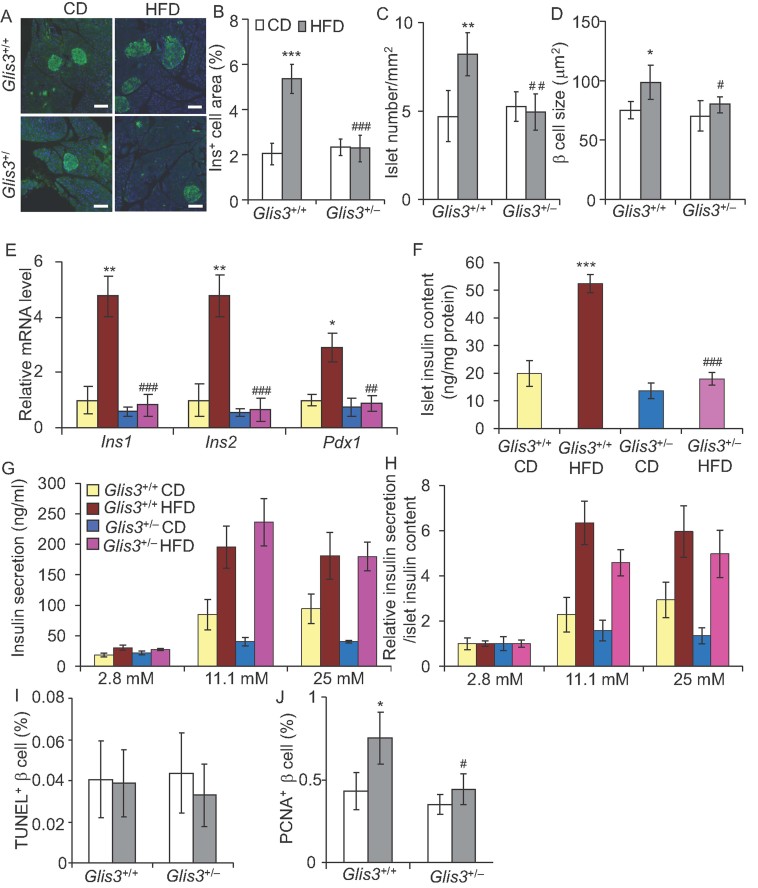
Impairment of beta cell mass expansion in *Glis3^+/−^* mice with HFD feeding **A,B.** Representative images of insulin immunostaining (**A**) and Ins^+^ cell area (***p = 0.00016 *versus Glis3*^*+/+*^ CD; ^###^*p* = 0.00013 *versus Glis3*^*+/+*^ HFD) (**B**) in the pancreas of *Glis3*^*+/+*^ and *Glis3*^*+/−*^ mice fed with a CD or HFD for 20 weeks. Scale bar, 100 µm.**C,D.** Islet density (**p = 0.009 *versus Glis3*^*+/+*^ CD; ^##^*p* = 0.003 *versus Glis3*^*+/+*^ HFD) (**C**) and mean beta cell size (*p = 0.014 *versus Glis3*^*+/+*^ CD; ^#^*p* = 0.027 *versus Glis3*^*+/+*^ HFD) (**D**) in the pancreas of *Glis3*^*+/+*^ and *Glis3*^*+/−*^ mice fed with a CD or HFD for 20 weeks.**E.** Relative mRNA expression of *Ins1*, *Ins2* and *Pdx1* (*p < 0.05, **p < 0.01, *versus Glis3*^*+/+*^ CD. ^##^*p* < 0.01, ^###^*p* < 0.001 *versus Glis3*^*+/+*^ HFD) in the pancreas of *Glis3*^*+/+*^ and *Glis3*^*+/−*^ mice fed with a CD or HFD for 20 weeks.**F–H.** Islet insulin content (**F**) (***p = 0.0002 *versus Glis3*^*+/+*^ CD; ^###^*p* = 0.00008 *versus Glis3*^*+/+*^ HFD), glucose-stimulated insulin secretion (GSIS) (**G**) and GSIS normalized to islet insulin content (**H**) in isolated islets of *Glis3*^*+/+*^ and *Glis3*^*+/−*^ mice fed with a CD or HFD for 20 weeks.**I,J.** TUNEL^+^ beta cell (**I**) and PCNA^+^ beta cell (**J**) (*p = 0.024 *versus Glis3*^*+/+*^ CD; ^#^*p* = 0.02 *versus Glis3*^*+/+*^ HFD), normalized to total beta cell number, in the pancreas of *Glis3*^*+/+*^ and *Glis3*^*+/−*^ mice fed with a CD or HFD for 20 weeks. Results were analyzed by student's *t*-test and presented as the mean ± standard error (S.E.).

Adult type 2 diabetes may be associated with failure of beta cell expansion (Ackermann & Gannon, 2007; Sachdeva & Stoffers, 2009; Weir & Bonner-Weir, 2004). To determine whether *Glis3* is required for beta cell expansion in response to HFD feeding, we examined pancreatic insulin positive cell area (indicating beta cell mass) in *Glis3*^+/−^ and *Glis3*^+/+^ mice fed a CD or HFD for 20 weeks. In *Glis3*^+/+^ mice, HFD induced >2.6-fold increase in beta cell mass compared to CD-fed mice, whereas *Glis3*^+/−^ mice on HFD or CD failed to show any difference in beta cell mass (Fig 2A and B). Whilst both islet density (Fig 2C) and the size of individual beta cells (Fig 2D, indicating hypertrophy) increased in *Glis3*^+/+^ mice with HFD feeding, these parameters remained unchanged in *Glis3*^+/−^ mice. As expected, the mRNA expression of pancreatic mature beta cell markers, such as *Ins1*, *Ins2* and *Pdx1* (Fig 2E), and islet immunoreactive insulin content (Fig 2F) were drastically reduced in the HFD-fed *Glis3*^+/−^ mice compared with *Glis3*^+/+^ mice. Therefore, *Glis3* is required for normal compensatory beta cell mass expansion in response to HFD feeding.

To examine whether *Glis3* haploinsufficiency affects insulin secretion, we quantified glucose-stimulated insulin secretion (GSIS) in isolated islets. GSIS showed no significant difference in insulin secretion from the islets of *Glis3*^+/−^ and *Glis3*^+/+^ mice fed a HFD, although the islets of *Glis3*^+/−^ showed a trend towards lower insulin secretion compared to those of *Glis3*^+/+^ mice under chow condition (Fig 2G). Importantly, we observed no significant defect of insulin secretion in the islets of *Glis3*^+/−^ mice, when the data were normalized to islet insulin content (Fig 2H).

### *Glis3* is required for beta cell proliferation via regulating *Ccnd2* transcription

**Figure 3:**
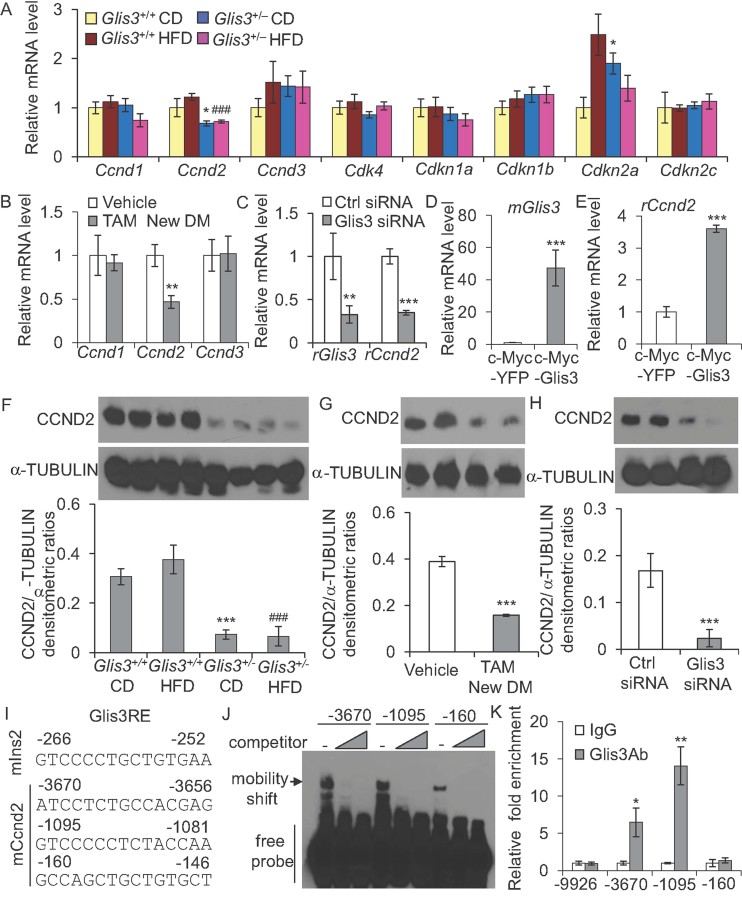
*Glis3* is required for beta cell proliferation and directly regulates *Ccnd2* transcription **A.** The mRNA expression of *Ccnd1*, *Ccnd2*, *Ccnd3*, *Cdk4*, *Cdkn1a*, *Cdkn1b*, *Cdkn2a* and *Cdkn2c* in the islets of *Glis3*^*+/+*^ and *Glis3*^*+/−*^ mice fed with a CD (*n* = 5) or HFD (*n* = 6) for 20 weeks. *p < 0.05, *versus Glis3*^*+/+*^ CD. ^#^*p* < 0.05, ^###^*p* < 0.001, *versus Glis3*^*+/+*^ HFD.**B.** The mRNA expression of *Ccnd1*, *Ccnd2* and *Ccnd3* in the islets of *Glis3*^*fl/fl*^*/Pdx1Cre*^*ERT+*^ mice treated with TAM (newly diabetic, plasma glucose just reaching 250 mg/dl) or vehicle. **p = 0.004 *versus* vehicle-treated mice. *N* = 6 for each group.**C.** The mRNA expression of *Glis3* and *Ccnd2* in INS-1 derived 832/13 cells transfected with control siRNA or *Glis3* siRNA for 48 h. **p = 0.003, ***p = 0.000007, *versus* control siRNA group. r: rat.**D,E.** The mRNA expression of *Glis3* **(D)** and *Ccnd2* **(E)** in INS-1 derived 832/13 cells stably overexpressing c-Myc-YFP or c-Myc-Glis3. ***p = 0.00002 and 0.0007 *versus* c-Myc-YFP group, respectively. m: mouse, r: rat.**F–H.** Representative Western blots of CCND2 and densitometri ratios of CCND2/alpha tubulin in the islets of *Glis3*^*+/+*^ and *Glis3*^*+/−*^ mice fed with a CD or HFD for 20 weeks **(F)** (***p = 0.00002 *versus Glis3*^*+/+*^ CD; ^###^*p* = 0.00008 *versus Glis3*^*+/+*^ HFD**)**, and in the islets of *Glis3*^*fl/fl*^*/Pdx1Cre*^*ERT+*^ mice treated with TAM (newly diabetic) or vehicle (G) (***p < 0.0001 *versus* vehicle-treated group), as well as in INS-1 derived 832/13 cells transfected with control siRNA or *Glis3* siRNA for 48 h **(H)** (*n* = 4, ***p = 0.0004 *versus* control siRNA group). Alpha tubulin was used as an internal control.**I.** Alignment of the 15-bp sequences (Glis3RE) located at −3670, −1095, and −160 in the 10-Kb mouse *Ccnd2* gene promoter. The Glis3RE in the mouse *Ins2* promoter was used as a comparison.**J.** EMSA using an *in vitro* translated GLIS3-ZFD peptide was performed with biotin-labeled probes containing putative Glis3REs sequences at −3670, −1095, and −160 in mouse *Ccnd2* gene promoter. Five- or fifty fold corresponding non-biotinylated Glis3REs were added as competitors. The specific band was indicated by an arrow.**K.** ChIP assays with anti-GLIS3 or IgG control were performed in the islets of wild type C57BL/6 mice. Immunoprecipitated DNA was purified and analyzed by qPCR using primers specifically spanning the putative Glis3RE region at −3690, −1095 and −160 sites and a control fragment located at −9926 of mouse *Ccnd2* promoter. Results were analyzed by student's *t*-test and presented as the mean ± S.E. from three independent experiments. *p = 0.048, **p = 0.007, *versus* IgG control.

Pancreatic beta cell mass expansion is a normal response to an increased demand for insulin, as occurs when mice are fed a HFD, total beta cell mass being modulated by cell proliferation and/or apoptosis (Ackermann & Gannon, 2007; Sachdeva & Stoffers, 2009). As we detected no difference in the number of apoptotic beta cells in *Glis3*^+/+^ and *Glis3*^+/−^ islets (Fig 2I), we examined beta cell proliferation as reflected by proliferating cell nuclear antigen (PCNA) staining and found that HFD feeding led to a significant increase in the number of PCNA^+^ beta cells in the islets of *Glis3*^+/+^ mice but no change in *Glis3*^+/−^ mice (Fig 2J). D-type cyclins, particularly cyclin D2 (*Ccnd2*) and D1, are essential for maintaining postnatal pancreatic beta cell mass (Georgia & Bhushan, 2004; Kushner et al, 2005). We therefore examined mRNA expression of D-type cyclins and other cell cycle-related genes in the isolated islets of these mice. qRT-PCR showed that the expression of *Ccnd2*, a predominant D-type cyclin in pancreatic beta cells (Georgia & Bhushan, 2004; Kushner et al, 2005) which was reported to be crucial for beta cell mass expansion (Georgia et al, 2010), was downregulated in the islets of *Glis3*^+/−^ mice compared to *Glis3*^+/+^ mice fed the same diets (Fig 3A); the expression of cyclin-dependent kinase inhibitor 2a (*Cdkn2a*) was upregulated in the islets of *Glis3*^+/−^ mice compared to *Glis3*^+/+^ mice fed regular chow; no difference in the expression of *Ccnd1*, *Ccnd3*, cyclin-dependent kinase 4 (*Cdk4*), *Cdkn1a*, *Cdkn1b or Cdkn2c* was detected (Fig 3A). We further confirmed by qRT-PCR that the mRNA expression of *Ccnd2* was downregulated in the islets of beta cell-specific *Glis3-*deficient mice (Fig 3B) and in *Glis3*-knockdown 832/13 cells (Fig 3C), while it was upregulated in *Glis3*-overexpressing 832/13 cells (Fig 3D and E). To corroborate these findings at the mRNA level, we found that at the protein level CCND2 was lower in the islets of *Glis3*^+/−^ mice fed either a chow or a HFD (Fig 3F) and in the islets of beta cell-specific *Glis3-*deficient mice (Fig 3G) as well as in *Glis3*-knockdown 832/13 cells (Fig 3H).

To determine whether GLIS3 directly regulates *Ccnd2* transcription, we searched in the mouse *Ccnd2* promoter for a Glis3RE that we recently uncovered in the insulin gene (5′-GTCCCCTGCTGTGAA-3′; Yang et al, 2009) and identified three putative Glis3RE sequences located at −3670, −1095 and −160, in the 10-kb promoter region (Fig 3I). We first performed EMSA using *in vitro*-translated DNA binding motif of GLIS3, the zinc finger domain (ZFD). The *in vitro*-translated GLIS3 (ZFD) was verified by [^35^S] labelling autoradiography (Supporting Information Fig S1). We found that GLIS3 (ZFD), but not two control proteins, red fluorescent protein (RFP) or dihydrofolate reductase (DHFR), binds to the Glis3RE at −3670, −1095 and −160, and the complexes were out-competed by molar excess of the corresponding non-biotinylated Glis3REs (Fig 3J, Supporting Information Fig S2). To further determine whether GLIS3 was bound to any of these sites *in vivo*, we performed GLIS3 ChIP assay in wild-type C57BL/6 islets. As shown in Fig 3K, GLIS3 occupies two of the Glis3REs, at −3670 and −1095 sites, in the mouse *Ccnd2* promoter. The discrepancy of site −160 between EMSA and ChIP assay data probably reflects the difference of *in vitro* and *in vivo* systems. These results are consistent with the interpretation that *Glis3* is required for the beta cell proliferative response in HFD-fed mice by directly regulating *Ccnd2* transcription.

### *Glis3* inactivation in adult pancreatic beta cells leads to severe diabetes in mice

**Figure 4:**
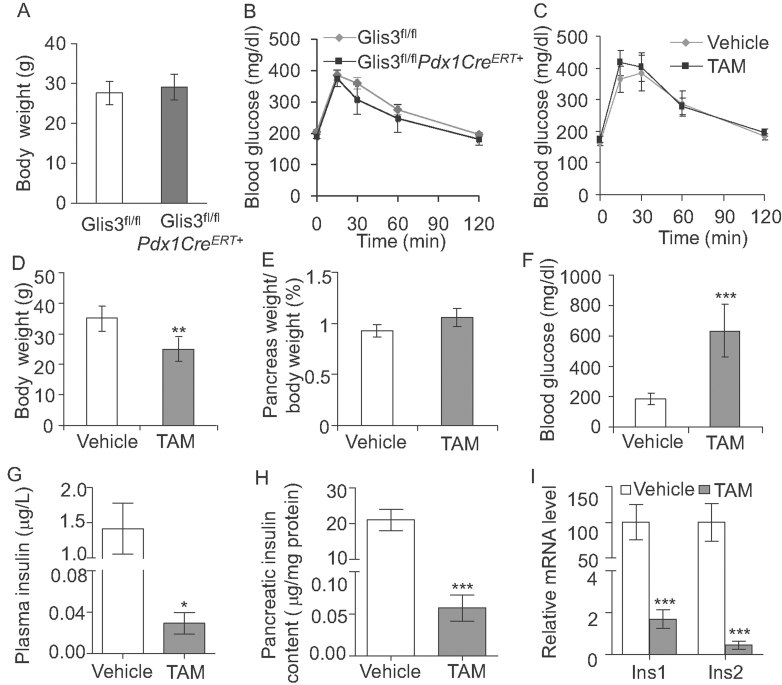
*Glis3* inactivation in adult beta cell leads to severe diabetes **A.** Body weights of 8-week old male mice of *Glis3*^*fl/fl*^ and *Glis3*^*fl/fl*^*/Pdx1Cre*^*ERT+*^ littermates before TAM treatment (*n* = 6).**B.** Gavage GTT was performed after 6 h fast. Plasma glucose was measured at time 0, 15, 30, 60 and 120 min after glucose injection (1.5 g/kg BW) in *Glis3*^*fl/fl*^ and *Glis3*^*fl/fl*^*/Pdx1Cre*^*ERT+*^ littermates before TAM treatment (*n* = 6).**C.** Eight-week old *Glis3*^*fl/fl*^ male mice were treated with TAM or vehicle (peanut oil with 10% ethanol). Eight weeks after treatment, gavage GTT was performed after 6 h fast (*n* = 5).**D–I.** *Glis3*^*fl/fl*^*/Pdx1Cre*^*ERT+*^ mice were treated with TAM or vehicle, and 8 weeks after treatment, their body weights (**D**) (**p = 0.0014 *vs.* vehicle-treated mice), pancreas weight/body weight ratio (**E**), blood glucose (**F**) (**p = 0.00005 *vs.* vehicle-treated mice), plasma insulin (**G**) (**p = 0.02 *vs.* vehicle-treated mice), pancreatic insulin content (**H**) (***p = 0.0002 *vs.* vehicle-treated mice), insulin (*Ins1*, *Ins2*) gene expression (**I**) (***p < 0.0001, vs. vehicle-treated mice) were shown (*n* = 6). Results were analysed by student's *t*-test and presented as the mean ± SE.

Whilst studies in adult *Glis3*^+/−^ mice indicate that impaired beta cell function and growth occur in the presence of HFD feeding, they do not address the important question whether *Glis3* is absolutely required for beta cell maintenance in the absence of environmental stress such as HFD. To examine the role of *Glis3* in normal dietary conditions, we intercrossed the conditionally targeted *Glis3*^*fl*/*fl*^ mice with the TAM regulatable *Pdx1Cre*^*ERT*+^ mice (Gannon et al, 2000) to produce *Glis3*^*fl*/*fl*^/*Pdx1Cre*^*ERT*+^ mice. These animals were born normal size and reached adulthood with normal blood glucose and without any sign of compromised health (Fig 4A and B). We administered TAM to 8-week old male *Glis3*^*fl*/*fl*^/*Pdx1Cre*^*ERT*+^ mice to induce a beta cell-specific deletion of the *Glis3* gene. Control mice (*Glis3*^*fl*/*fl*^) were healthy and euglycaemic before TAM treatment, and remained so 8 weeks after treatment (Fig 4C). In contrast, *Glis3*^*fl*/*fl*^/*Pdx1Cre*^*ERT*+^ mice, which had been euglycaemic before treatment (Fig 4B), developed diabetes within 2–4 weeks of TAM injection, whilst vehicle-treated *Glis3*^*fl*/*fl*^/*Pdx1Cre*^*ERT*+^ mice remained euglycaemic. The physical condition of TAM treated *Glis3*^*fl*/*fl*^/*Pdx1Cre*^*ERT*+^ mice deteriorated with time. Eight weeks after treatment they displayed significantly lower body weight (Fig 4D) and severe hyperglycaemia (>600 mg/dl; Fig 4F), whilst their plasma insulin was almost completely absent (Fig 4G). Furthermore, the pancreatic insulin content and mRNA level in the TAM-treated *Glis3*^*fl*/*fl*^/*Pdx1Cre*^*ERT*+^ mice were <1% than those of vehicle-treated *Glis3*^*fl*/*fl*^/*Pdx1Cre*^*ERT*+^ controls (Fig 4H and I), indicating that TAM induced beta cell-specific *Glis3* deletion caused massive loss of insulin expression in these mice.

**Figure 5:**
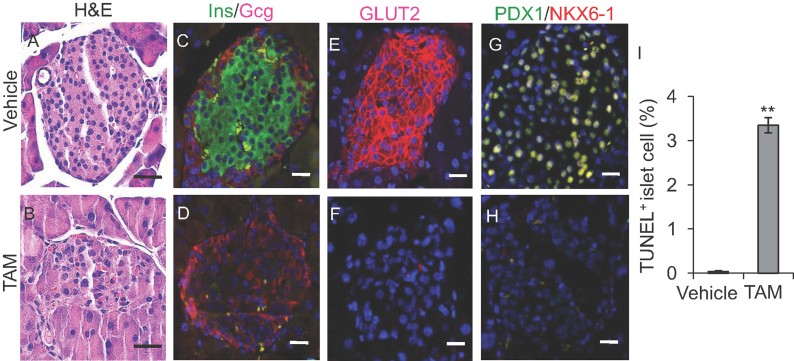
Islet histology and immunostaining of mature beta cell markers in *Glis3^fl/fl^/Pdx1Cre^ERT+^* mice eight weeks after TAM or vehicle treatment **A,B.** Representative islet histology in the pancreas of *Glis3*^*fl/fl*^*/Pdx1Cre*^*ERT+*^ mice 8 weeks after TAM or vehicle administration. Scale bar, 20 µm.**C–H.** Immunostaining of Ins, Gcg, GLUT2, PDX1 and NKX6-1 in the pancreas of *Glis3*^*fl/fl*^*/Pdx1Cre*^*ERT+*^ mice 8 weeks after TAM or vehicle administration. Scale bar, 20 µm (except for panel **G**, **H**: 25 µm).**I.** Percentage of TUNEL^+^ islet cell/total islet cells (**p = 0.002 *versus* vehicle-treated mice) in the pancreas of *Glis3*^*fl/fl*^*/Pdx1Cre*^*ERT+*^ mice 8 weeks after TAM or vehicle administration. Result was analyzed by student's *t*-test and presented as the mean ± S.E.

Eight weeks after treatment, the ratio of pancreas weight to body weight was similar (Fig 4E) between vehicle and TAM-treated *Glis3*^*fl*/*fl*^/*Pdx1Cre*^*ERT*+^ mice. Histological examination of the pancreas revealed a markedly reduced islet density in the TAM-treated *Glis3*^*fl*/*fl*^/*Pdx1Cre*^*ERT*+^ mice; the residual islets were small and severely disorganized (Fig 5A and B). In agreement with the markedly reduced insulin mRNA, immunostaining showed a near total absence of insulin-positive cells among the residual islets as compared to vehicle-treated mice (Fig 5C and D). We next investigated the expression of other mature beta cell markers GLUT2 (Pang et al, 1994; Thorens et al, 1990), PDX1 (Jonsson et al, 1994; Offield et al, 1996; Ohlsson et al, 1993) and NKX6-1 (Jensen et al, 1996; Oster et al, 1998; Rudnick et al, 1994) by immunostaining and found that all three mature beta cell marker-expressing cells were markedly decreased in the islets of TAM-treated *Glis3*^*fl*/*fl*^/*Pdx1Cre*^*ERT*+^ mice, as compared to vehicle-treated controls (Fig 5E–H, Supporting Information Fig S3). To determine if increased apoptosis contributed to beta cells loss, we performed terminal deoxynucleotidyl transferase dUTP nick end labelling (TUNEL) assay and immunostaining of cleaved caspase-3, a marker of apoptosis, and found that the number of TUNEL^+^ islet cells (Fig 5I) and cleaved caspase-3-positive cells (Supporting Information Fig S4), was greatly increased in the *Glis3*^*fl*/*fl*^/*Pdx1Cre*^*ERT*+^ mice 8 weeks after TAM treatment as compared to the vehicle-treated mice.

### *Glis3* maintains beta cell function by controlling insulin gene expression, but not beta cell apoptosis, in adult pancreas

**Figure 6:**
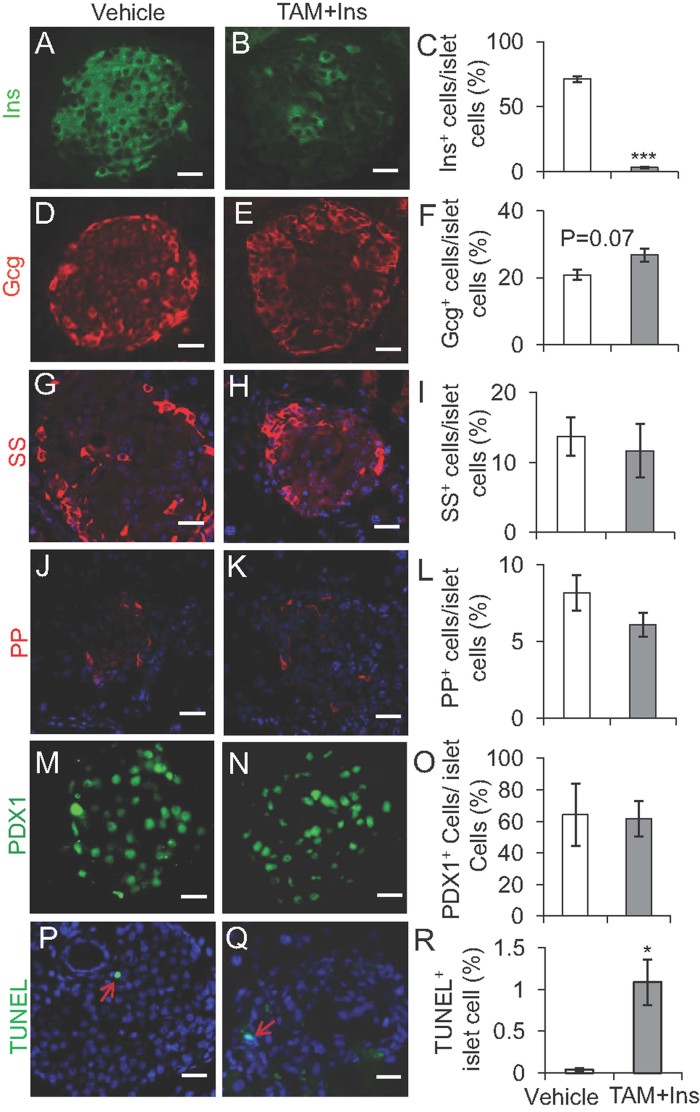
The expression of insulin, but not PDX1, is reduced in *Glis3^fl/fl^/Pdx1Cre^ERT+^* mice eight weeks after TAM plus insulin pellet treatments **A–L.** Representative immunostaining images and quantification of endocrine hormones such as Ins, Gcg, SS and PP in the pancreas of *Glis3*^*fl/fl*^*/Pdx1Cre*^*ERT+*^ mice eight weeks after TAM and implanted insulin pellets (blood glucose < 300 mg/dl) or vehicle administration. Results were analyzed by student's *t*-test and presented as the mean ± S.E. **p < 0.00001 *versus* vehicle-treated mice. Scale bar, 20 µm.**M–O.** Representative immunostaining images and quantification of PDX1 in the pancreas of *Glis3*^*fl/fl*^*/Pdx1Cre*^*ERT+*^ mice eight weeks after TAM and implanted insulin pellets or vehicle administration. Scale bar, 25 µm.**P–R.** TUNEL assay and TUNEL^+^ cell quantification in the pancreas of *Glis3*^*fl/fl*^*/Pdx1Cre*^*ERT+*^ mice eight weeks after TAM and implanted insulin pellets or vehicle administration. *p = 0.019 *versus* vehicle-treated mice. Scale bar, 20 µm. Note: In vehicle-treated mice, only one TUNEL^+^ islet cell was found among more than 50 islets.

The findings in Figs 4D–I and 5 were obtained in severely hyperglycaemic TAM-treated *Glis3*^*fl*/*fl*^/*Pdx1Cre*^*ERT*+^ mice, and some of the changes observed could be the consequence of glucotoxicity (Olson et al, 1993; Poitout & Robertson, 2002, 2008; Robertson et al, 2004). To address this possibility, we implanted insulin pellets to maintain the random blood glucose level to <300 mg/dl in the TAM-treated *Glis3*^*fl*/*fl*^/*Pdx1Cre*^*ERT*+^ mice. We then performed immunofluorescence staining and observed a more than 95% reduction in insulin staining in the islets of *Glis3*^*fl*/*fl*^/*Pdx1Cre*^*ERT*+^ mice 8 weeks after TAM administration in the insulin pellet-implanted mice (Fig 6A–C) as compared to vehicle-treated mice. In contrast, the levels of glucagon, somatostatin, PP and PDX1 were comparable between the two groups (Fig 6D–O). The TUNEL^+^ islet cells (Fig 6P–R) and cleaved caspase-3 staining (Supporting Information Fig S4) were, however, still higher in the islets of TAM-treated and insulin pellets implanted *Glis3*^*fl*/*fl*^/*Pdx1Cre*^*ERT*+^ mice whilst no difference of PCNA staining was noted, in comparison to vehicle-treated mice.

**Figure 7:**
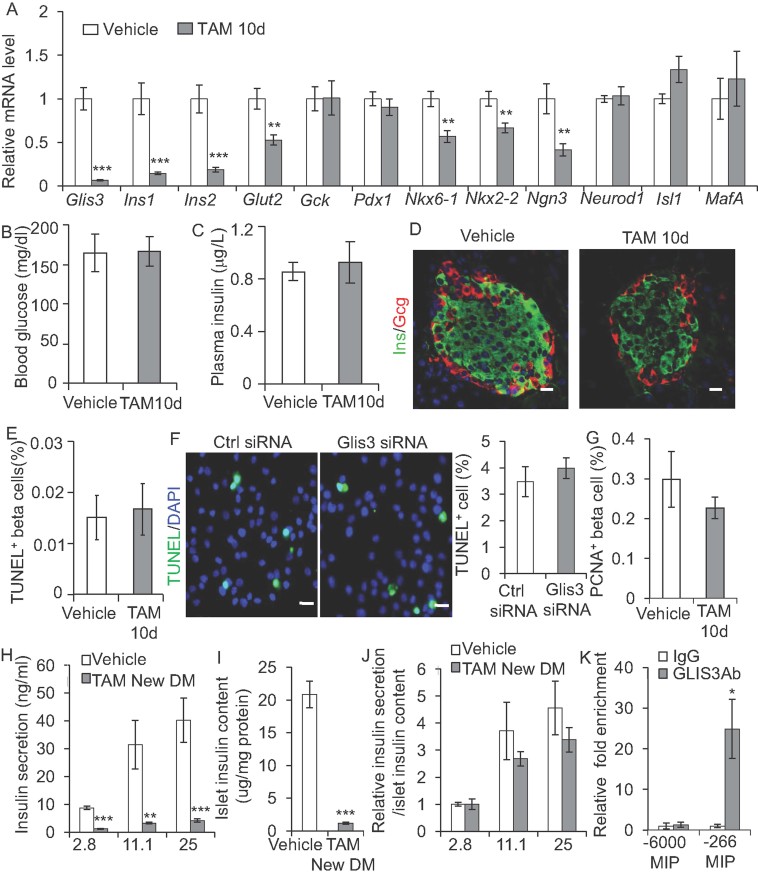
*Glis3* inactivation in adult beta cell reduces insulin expression independent of glucotoxicity **A.** The mRNA expression of *Glis3*, *Ins1*, *Ins2*, *Glut2* and *Gck* and islet enriched transcription factors (*Pdx1*, *Nkx6-1*, *Nkx2-2*, *Ngn3*, *Neurod1*, *Isl1* and *MafA*) in handpicked islets of *Glis3*^*fl/fl*^*/Pdx1Cre*^*ERT+*^ mice 10 days after TAM or vehicle administration (*n* = 6). Results were analyzed by student's *t*-test and presented as the mean ± S.E. **p < 0.01, ***p < 0.001, *versus* vehicle-treated mice.**B,C.** Blood glucose (**B**) and plasma insulin (**C**) in *Glis3*^*fl/fl*^*/Pdx1Cre*^*ERT+*^ mice 10 days after TAM or vehicle administration.**D.** Immunoreactive Ins and Gcg in *Glis3*^*fl/fl*^*/Pdx1Cre*^*ERT+*^ mice 10 days after TAM or vehicle administration. Scale bar, 20 µm.**E.** TUNEL^+^ beta cells, normalized to total islet cells in *Glis3*^*fl/fl*^*/Pdx1Cre*^*ERT+*^ mice 10 days after TAM or vehicle administration.**F.** Representative images and percentage of TUNEL^+^ cells in INS-1 derived 832/13 cells transfected with control siRNA or *Glis3* siRNA for 48 h. Scale bar, 20 µm.**G.** PCNA^+^ beta cells, normalized to total islet cells, in the pancreas of 10 days TAM or vehicle-treated *Glis3*^*fl/fl*^*/Pdx1Cre*^*ERT+*^ mice.**H–J.** Glucose-induced insulin secretion (GSIS) **(H)**, islet insulin content **(I)** and the relative GSIS/islet insulin content **(J)** in *Glis3*^*fl/fl*^*/Pdx1Cre*^*ERT+*^ mice treated with TAM (newly diabetic) or vehicle (*n* = 6). **p < 0.01, ***p < 0.001 *versus* vehicle-treated mice.**K.** ChIP assays with anti-GLIS3 or IgG control were performed in the islets of wild type C57BL/6 mice. Immunoprecipitated DNA was purified and analyzed by qPCR using primers specifically spanning the Glis3RE region (−266 MIP) of mouse insulin 2 promoter, the distal region (−6000 MIP) was used as a negative control. Results were analyzed by student's *t*-test and presented as the mean ± S.E. from three independent experiments. *p = 0.03 *versus* IgG control.

As the glycaemic control in the insulin implanted TAM-treated *Glis3*^*fl*/*fl*^/*Pdx1Cre*^*ERT*+^ mice was less than perfect, it was not possible to rule out glucotoxicity-related insulin reduction and beta cell apoptosis in these mice. To further explore the role of *Glis3* in the adult pancreas, we collected pancreata from TAM-treated *Glis3*^*fl*/*fl*^/*Pdx1Cre*^*ERT*+^ mice 10 days after TAM administration. Given that GLIS3 antibodies for are not available for immunocytochemical staining, we have determined the efficiency of *Glis3* deletion in isolated islets by q-RT-PCR, which showed that *Glis3* mRNA was reduced over 90% in the islets 10 days after TAM administration (Fig 7A). It should be noted that qRT-PCR underestimates *Pdx1*-Cre mediated *Glis3* silencing in beta cells because *Glis3* mRNA is also present in the non-beta islet cells (Senee et al, 2006). We further confirmed the efficiency of *Glis3* deletion in the pancreatic islets by *in situ* hybridization 10 days after TAM or vehicle administration (Supporting Information Fig S5). At this early time point, the mice still displayed euglycaemia (Fig 7B) and a tendency towards glucose intolerance (Supporting Information Fig S6), in the presence of normal plasma insulin (Fig 7C). We observed that the mRNA expression of *Pdx1*, a mature beta cell marker (Jonsson et al, 1994; Offield et al, 1996; Ohlsson et al, 1993), was unchanged in the islets isolated from the two groups (Fig 7A). Importantly, the level of insulin mRNA (Fig 7A) and immunoreactive insulin (Fig 7D) was significantly decreased at this early stage of TAM treatment as compared with vehicle treatment in *Glis3*^*fl*/*fl*^/*Pdx1Cre*^*ERT*+^ mice. However, the number of apoptotic islet cells reflected by TUNEL assay was not different between the two groups (Fig 7E). To corroborate the absence of a difference in the very low percentage of TUNEL^+^ cells in the pancreatic islets of TAM and vehicle-treated *Glis3*^*fl*/*fl*^/*Pdx1Cre*^*ERT*+^ mice, we next performed TUNEL assay in *Glis3*-knockdown INS-1 derived 832/13 cells and again observed no difference in TUNEL-positive cells between control siRNA and *Glis3* siRNA transfected cells (Fig 7F). Therefore, impairment in insulin expression at the mRNA and protein levels occurs early in TAM-treated *Glis3*^*fl*/*fl*^/*Pdx1Cre*^*ERT*+^ mice, an effect that precedes the onset of hyperglycaemia and the apoptosis that the latter induced.

Since *Glis3* is necessary for beta cell mass expansion in response to HFD feeding (Fig 2), we examined beta cell proliferation using PCNA immunostaining and found that there was no significant difference in the number of PCNA^+^ cells in islets isolated from vehicle and TAM-treated *Glis3*^*fl*/*fl*^/*Pdx1Cre*^*ERT*+^ mice (Fig 7G). Furthermore, the proportion of BrdU^+^ cells in *Glis3*-knockdown and control 832/13 cells was also similar (Supporting Information Fig S7). Therefore, sustained *Glis3* and *Ccnd2* expression is required for HFD-induced beta cell proliferation, but not for beta cell proliferation under basal conditions. GSIS analysis revealed decreased insulin release in islets isolated from newly diabetic *Glis3*^*fl*/*fl*^/*Pdx1Cre*^*ERT*+^ mice after TAM administration, compared to those from vehicle-treated mice (Fig 7H). However, no defect in insulin secretion was observed in the beta cell-specific *Glis3* deficient mice when normalized to islet insulin content (Fig 7I and J).

We previously identified a Glis3 response element (Glis3RE) in the insulin gene promoter and showed that *Glis3* directly and indirectly activates insulin gene transcription (Yang et al, 2009). To further explore whether GLIS3 binds to the insulin promoter *in vivo*, we performed ChIP assay on the islets of wild type C57BL/6 mice. The results revealed that GLIS3 binds to the endogenous proximal region (−266 MIP), but not a distal region (−6000 MIP; Fig 7K), of the mouse insulin 2 promoter. These observations further corroborate the conclusion that GLIS3 directly binds to the insulin promoter and regulates its expression. Therefore, the data in the induced beta cell-specific *Glis3*-deficient mouse model further highlight the key role of *Glis3* in controlling insulin gene expression in the adult pancreas *in vivo*.

Interestingly, the mRNA expression of *Glut2*, *Nkx6-1*, *Nkx2-2* and *Ngn3* was significantly downregulated at this early stage of TAM treatment as compared with vehicle treatment in *Glis3*^*fl*/*fl*^/*Pdx1Cre*^*ERT*+^ mice, whilst no change in the expression of *Gck*, *NeuroD1*, *Isl1* and *MafA* was detected (Fig 7A). The reduction of *Ngn3* mRNA in the adult islets of beta cell-specific *Glis3* deficient mice (Fig 7A) suggests that *Ngn3* may have contributed to *Glis3*'s effects on adult beta cell function. We note, however, that loss of *Ngn3* in adult mice was reported to cause relatively subtle effects on beta cell function in adult mice (Wang et al, 2009). Thus, given the very robust direct transactivation of *Glis3* on the insulin gene in the adult pancreas, these results indicate that *Ngn3* is unlikely to play a major role in the precipitous loss of insulin production and the resultant fulminant diabetes that occurs after *Glis3* deletion in the adult beta cells.

## DISCUSSION

In the present study, we have examined the function of *Glis3* in pancreatic beta cells in adult animals. *Glis3*^−/−^ mice die neonatally (Kang et al, 2009; Watanabe et al, 2009; Yang et al, 2011), making it difficult to determine the function of *Glis3* in the fully mature adult pancreas. We therefore generated two independent adult mouse models. First, we focused on heterozygous *Glis3*^+/−^ mice subjected to HFD feeding. Our results showed that *Glis3* is required for compensatory beta cell expansion in response to the HFD. The failure to expand beta cell mass in HFD-fed *Glis3*^+/−^ mice is primarily due to a decreased proliferation rate. Pancreatic beta cell replication is tightly controlled by multiple molecules (Ackermann & Gannon, 2007; Cozar-Castellano et al, 2006; Sachdeva & Stoffers, 2009). Among them, D-type cyclins, particularly *Ccnd2*, are essential for postnatal pancreatic beta cell growth (Georgia & Bhushan, 2004; Kushner et al, 2005) and compensatory expansion in response to insulin resistance (Georgia et al, 2010). Interestingly, we found that the mRNA expression of *Ccnd2* was downregulated in the islets of *Glis3*^+/−^ mice and beta cell-specific *Glis3* deficient mice. Furthermore, we provided evidence that GLIS3 directly binds to mouse *Ccnd2* promoter and regulates its transcription. These results demonstrate that *Glis3* is required for beta cell proliferation and mass expansion in response to HFD feeding at least partly by regulating *Ccnd2* transcription.

Second, to gain insight into whether *Glis3* is absolutely required for normal beta cell function in adult animals, we generated *Glis3*^*fl*/*fl*^/*Pdx1Cre*^*ERT*+^ mice for analysis. Treatment of these mice with TAM produces beta cell-specific inactivation of *Glis3* in adult animals, which led to fulminant diabetes. Pancreatic insulin mRNA, immunoreactive insulin content and plasma insulin level became essentially undetectable in these severely diabetic mice.

As hyperglycaemia *per se* has been reported to downregulate insulin expression as well as stimulate beta cell apoptosis (Olson et al, 1993; Poitout & Robertson, 2002, 2008; Robertson et al, 2004), we used two strategies to circumvent these effects of hyperglycaemia in the TAM-treated *Glis3*^*fl*/*fl*^/*Pdx1Cre*^*ERT*+^ mice. First, using insulin pellets we partially attenuated the hyperglycaemia in TAM-treated *Glis3*^*fl*/*fl*^/*Pdx1Cre*^*ERT*+^ mice to <300 mg/dl, but immunoreactive insulin was still greatly reduced in the islets of these mice, whilst the rate of beta cell apoptosis was still significantly higher than that in the vehicle-treated control mice. To circumvent the confounding effect of the residual mild to moderate hyperglycaemia in these mice, we next examined the TAM-treated *Glis3*^*fl*/*fl*^/*Pdx1Cre*^*ERT*+^ mice early at 10 days after TAM administration, when the animals were still euglycaemic and had normal plasma insulin. At this early time point, insulin mRNA level and immunoreactive insulin expression were already decreased. Importantly, in the absence of hyperglycaemia, the number of apoptotic cells in the pancreatic islets was not different between vehicle and TAM-treated mice, which was corroborated in *Glis3*-knockdown INS-1 derived 832/13 cells, indicating that the increased apoptosis that occurred after onset of hyperglycaemia was a consequence of glucotoxicity and not a direct effect of loss of *Glis3* expression.

We showed recently that *Glis3* is a potent transactivator of the insulin promoter (Yang et al, 2009); it physically and functionally interacts with PDX1, MAFA and NEUROD1 to modulate insulin promoter activity (Yang et al, 2009). Here, we used conditional *Glis3*-deficient mouse models to demonstrate the pivotal function of *Glis3* in controlling insulin gene expression in the adult pancreas *in vivo*. It is important to note that, to evaluate insulin secretion in *Glis3*-deficient mice, one must take into consideration the fact that *Glis3* is a potent insulin gene transactivator (Yang et al, 2009) and *de novo* insulin biosynthesis and insulin content of islets are markedly reduced in Glis3-deficient mice. We, therefore, normalized GSIS to islet insulin content, a strategy commonly used by other investigators in the field (Gu et al, 2010; Preitner et al, 2004), and found no significant difference in GSIS of wild-type islets *versus Glis3*-deficient islets.

Whilst *Glis3* is upstream of *Ngn3* in the foetal pancreas and loss of *Glis3* during embryonic development produces neonatal diabetes, a consequence of defective *Ngn3*-mediated islet differentiation (Yang et al, 2011), in the adult animal, *Ngn3* appears to play little or no role in the diabetogenic effect of loss of *Glis3*. Loss of *Glis3* directly causes drastically reduced insulin expression, leading to hyperglycaemia which subsequently induces beta cell apoptosis probably via glucotoxicity, triggering a vicious cycle that culminates in severe fulminant diabetes.

Taken together, our studies provide a molecular basis for the *GLIS3* locus conferring susceptibility to type 1 (Barrett et al, 2009) and type 2 diabetes (Boesgaard et al, 2010; Dupuis et al, 2010; Liu et al, 2011), both of which involve defects in beta cell function (in the presence of insulin resistance in the case of the type 2 disease). We found that *Glis3* is absolutely required for normal insulin gene expression in adult beta cells *in vivo*. Furthermore, reduced expression of *Glis3* leads to impaired HFD-induced beta cell expansion. Thus, in addition to its pivotal role in foetal pancreatic islet differentiation (Kang et al, 2009; Watanabe et al, 2009; Yang et al, 2011), *Glis3* is also a key beta cell transcription factor that is essential for normal beta cell function and mass maintenance during adulthood.

## MATERIALS AND METHODS

### Generation of islet beta cell-specific *Glis3* targeted mice

*Glis3*^*fl*/*fl*^ and *Glis3*^+/−^ mice have been generated as described previously (Yang et al, 2011). *Glis3*^+/+^ and *Glis3*^+/−^ male mice were randomized to receive either regular chow or a HFD containing 40% kcal fat (TestDiet, 5TFH) at 4 weeks of age. *Pdx1Cre*^*ERT*+^ mice were kindly provided by Dr. Maureen Gannon (Vanderbilt University, Nashville, TN; Gannon et al, 2000). We crossed *Glis3*^*fl*/*fl*^ mice with *Pdx1Cre*^*ERT*+^ mice to obtain *Glis3*^*fl*/*fl*^/*Pdx1Cre*^*ERT*+^ mice. To activate Cre^ERT^ nuclear localization and *Glis3* deletion in pancreatic beta cells, we administrated TAM (Sigma–Aldrich, St. Louis, MO, USA; dissolved in peanut oil with 10% ethanol) to 8-week-old male mice intraperitoneally at a dose of 3 mg/mouse/day for 7 consecutive days.

In some experiments, we implanted sustained-release insulin pellet (Linshin Canada, Inc., Ontario, Canada) to TAM-treated *Glis3*^*fl*/*fl*^/*Pdx1Cre*^*ERT*+^ mice subcutaneously near the cervical region once the random glucose reached 300 mg/dl or higher. Animals were considered newly diabetic when blood glucose measurement reached 250 mg/dl. All animal studies were performed using protocols approved by the Institutional Animal Care and Use Committee (IACUC) at Baylor College of Medicine.

**The paper explained**

PROBLEM:

Genome-wide association studies identified *GLIS3* as a susceptibility locus for type 1 and type 2 diabetes in adult populations, but the underlying mechanism remains unknown. Genetic inactivation of *Glis3* by gene targeting (*Glis3*^−/−^) in mice was shown to lead to neonatal diabetes. However, the fact that *Glis3*^−/−^ mice die neonatally makes it a challenge to investigate the functional role of *Glis3* in the adult pancreas.

RESULTS:

To explore *Glis3*'s role in adults, we generated two independent mouse models: mice with haploinsufficiency of *Glis3* (*Glis3*^+/−^) and inducible pancreatic beta cell-specific *Glis3* deficiency (*Glis3*^*fl/fl*^*/Pdx1Cre*^*ERT+*^). *Glis3*^+/−^ mice develop HFD-induced diabetes because of impaired beta cell mass expansion. Ccnd2, a predominant D-type cyclin in pancreatic beta cells, has been reported to be crucial for beta cell mass expansion after HFD feeding. Here we provide evidence that GLIS3 controls beta cell proliferation in response to high fat feeding at least partly by regulating *Ccnd2* transcription. Adult *Glis3*^*fl/fl*^*/Pdx1Cre*^*ERT+*^ mice are euglycaemic. TAM-mediated beta cell-specific inactivation of *Glis3* in adult mice acutely downregulates insulin gene expression, leading to hyperglycaemia and subsequently enhanced beta cell apoptosis.

IMPACT:

Our findings are directly relevant to the control of adult pancreatic beta cell function and mass. There is an ongoing diabetes epidemic worldwide. The discoveries reported herein have public health implications in view of recent GWAS analyses that document the association of the *GLIS3* locus with type 1 and type 2 diabetes in adult human populations. The study has enhanced our understanding of the mechanisms that underlie the development of diabetes in association with diet-induced obesity. Furthermore, we have identified GLIS3 as a potential new therapeutic target for beta cell mass expansion to treat diabetes.

### Cell culture and siRNA transfection

Rat INS-1 derived 832/13 insulinoma cells (gift of Dr. Christopher Newgard, Duke University) were maintained as described (Hohmeier et al, 2000). We used Lipofectamine 2000 (Invitrogen) for transfection according to the manufacturer's instructions. The c-Myc-Glis3 cDNAs as well as control yellow fluorescent protein (YFP) were amplified by using PCR and cloned into the retroviral vector MSCV (Clontech, Mountain View, CA).

For siRNA experiments, all control and *Glis3* siRNAs were synthesized by Dharmacon RNAi Technologies (Thermo Fisher Scientific, Waltham, MA). The rat Glis3 targeting siRNA sequences were: sense, 5′-GCAUCACAGUGUACGAUUUUU-3′; antisense, 5′-AAAUCGUACACUGUGAUGCUU-3′ (Yang et al, 2009). DharmaFECT siRNA transfection reagent 2 was used for delivering siRNA into target cells. For BrdU staining, 832/13 cells were seeded on Collagen I Coated Coverslips (BD, Franklin Lakes, NJ) and transfected with siRNA; 2 h before harvest, BrdU (10 µM, Sigma) was added to the cultures, and cells were fixed in 1% paraformaldehyde and stained for BrdU using rat monoclonal anti-BrdU antibody (Abcam, Cambridge, MA).

### Gavage glucose tolerance test (GTT) and insulin measurement

Mice were fasted for 6 h and then delivered d-glucose (1.5 g/kg body weight) into the stomachs by a gavage needle. Glucose levels were measured at 0, 15, 30, 60 and 120 min post-gavage using One Touch Glucometer (Lifescan, Milpitas, CA, USA). We measured plasma insulin as described previously (Yang et al, 2011).

### Immunofluorescence staining

We performed immunostaining on the paraffin embedded sections as described previously (Yang et al, 2009, 2011). In some experiments, apoptosis was determined by direct TUNEL labelling assay using *In Situ* Cell Death Detection Kit, Fluorescein (Roche Applied Science, Indianapolis, IN, USA) following the manufacturer's instructions.

### Insulin-positive cell quantification

We estimated insulin-positive cell area (indicating beta cell mass) using ImageJ 1.4 (NIH, Bethesda, MD, USA) on six fluorescent sections of pancreatic islets (approximately every tenth section) that had been processed from four independent pancreata (Yang et al, 2011). Mean beta cell size was calculated by measuring Ins^+^ cell area and number of beta cell per section for at least six sections (approximately every tenth section) from four mice for each group using ImageJ 1.4 software (NIH). PCNA^+^ and TUNEL^+^ beta cells were counted on 6–12 sections (approximately every tenth section) processed from four independent animals and showed as the percentage of the total number of beta cells.

### *In situ* hybridization

We performed non-radioactive *in situ* hybridization with digoxigenin-UTP (Roche Diagnostics Corporation) labelled antisense RNA probes using a 460 bp (NM_175459, 1174-1633) mouse *Glis3* cDNA clone, with the help of the RNA *In Situ* Hybridization Core at Baylor College of Medicine.

### RNA isolation and quantitative polymerase chain reaction (qPCR)

Islet isolations from mouse pancreata were performed using collagenase P (Roche) digestion. We used TRIzol (Invitrogen, Carlsbad, California, USA) or Mini RNA Isolation I Kit (Zymo Research, Irvine, CA, USA) for total RNA isolation and the RNA was treated with amplification grade DNase I (Invitrogen) to remove genomic DNA contamination. Reverse transcription and qPCR were performed as described previously (Yang et al, 2009, 2011). Primer sequences are showed in Supporting Information Table S1.

### Glucose-stimulated insulin secretion (GSIS)

We cultured islets overnight in 11.1 mM glucose RPMI 1640 media. After washing the islets twice in fresh KRB buffer plus 1% BSA, we sequentially incubated 15 islets of similar size from different group of mice in KRB buffer with 1% BSA and 2.8, 11.1 or 25 mM glucose for 1 h at 37°C. After each incubation, buffer was removed and frozen at −80°C until insulin determination by ELISA (Mercodia, Winston Salem, NC). We then washed the islets three times with PBS and sonicated them in 0.5 ml of 0.2 N HCl with 70% ethanol. Islet insulin content was measured as described previously (Yang et al, 2011).

### Western blotting

We sonicated and boiled islet samples in a lysis solution containing 1% SDS, and determined the protein concentration by Protein Assay Dye Reagent Concentrate (Bio-Rad, Hercules, CA). Thirty micrograms of protein per islet sample (pooled from 2 to 4 mice) were separated by 10% SDS–PAGE, blotted to nitrocellulose membrane (Bio-Rad), and incubated either with mouse cyclin D2 Ab-2 antibody (1:400 dilution, Thermo Fisher Scientific) or with mouse anti-alpha-tubulin antibody (1:5000 dilution). After subsequent incubation with goat anti-mouse IgG horseradish peroxidase-conjugated antibody (Bio-Rad), the membranes were developed using enhanced chemiluminescence. Quantitation was performed using a Gel-Pro Analyzer (Media Cybernetics, Rockville, MD).

### Electrophoretic mobility shift assay (EMSA)

The GLIS3 ZFD (484–687 aa), and two negative control proteins RFP and DHFR (New England Biolabs, Ipswich, MA) were *in vitro* translated using TnT® T7 Quick Coupled Transcription/Translation System (Promega, Madison, WI, USA). L-Methionine [^35^S] (MP Biomedicals, Solon, OH) was used for labelling autoradiography. The translated peptide GLIS3(ZFD) was used for EMSA as described previously (Yang et al, 2009). Assays were conducted using a biotin-labelled double-stranded oligonucleotides probe containing the recognition sequence for GLIS3 (Supporting Information Table S1). Unlabelled wild type Glis3RE double stranded oligonucleotides were added as competitors.

### Chromatin immunoprecipitation (ChIP)

We performed ChIP assays as described previously (Yang et al, 2011) with minor modifications. Islets were isolated from adult C57BL/6 mice and immediately snap frozen in liquid nitrogen and stored at −80°C until use. Frozen islets were pooled and thawed on ice and crosslinked immediately in 1.5% formaldehyde at room temperature for 15 min. Following quenching in 125 mM glycine, samples were resuspended in cold 1× PBS. We incubated the sheared preparations (∼400 islets for each ChIP reaction) with anti-GLIS3 antibody (Yang et al, 2011) overnight at 4°C. After removing the protein and purifying the DNA, we performed qPCR analyses to detect the Glis3RE fragment. Primer sequences are showed in Supporting Information Table S1.

### Statistical analysis

The standard Student's 2-tailed *t* test was used for other comparisons. Results are presented as the mean ± standard error (SE).

## Supplementary material

Detailed facts of importance to specialist readers are published as ”Supporting Information”. Such documents are peer-reviewed, but not copy-edited or typeset. They are made available as submitted by the authors.

## References

[b1] Ackermann AM, Gannon M (2007). Molecular regulation of pancreatic beta-cell mass development, maintenance, and expansion. J Mol Endocrinol.

[b2] Barker A, Sharp SJ, Timpson NJ, Bouatia-Naji N, Warrington NM, Kanoni S, Beilin LJ, Brage S, Deloukas P, Evans DM (2011). Association of genetic Loci with glucose levels in childhood and adolescence: a meta-analysis of over 6,000 children. Diabetes.

[b3] Barrett JC, Clayton DG, Concannon P, Akolkar B, Cooper JD, Erlich HA, Julier C, Morahan G, Nerup J, Nierras C (2009). Genome-wide association study and meta-analysis find that over 40 loci affect risk of type 1 diabetes. Nat Genet.

[b4] Boesgaard TW, Grarup N, Jorgensen T, Borch-Johnsen K, Hansen T, Pedersen O (2010). Variants at DGKB/TMEM195, ADRA2A, GLIS3 and C2CD4B loci are associated with reduced glucose-stimulated beta cell function in middle-aged Danish people. Diabetologia.

[b5] Cho YS, Chen CH, Hu C, Long J, Ong RT, Sim X, Takeuchi F, Wu Y, Go MJ, Yamauchi T (2012). Meta-analysis of genome-wide association studies identifies eight new loci for type 2 diabetes in east Asians. Nat Genet.

[b6] Cozar-Castellano I, Fiaschi-Taesch N, Bigatel TA, Takane KK, Garcia-Ocana A, Vasavada R, Stewart AF (2006). Molecular control of cell cycle progression in the pancreatic beta-cell. Endocr Rev.

[b7] Dupuis J, Langenberg C, Prokopenko I, Saxena R, Soranzo N, Jackson AU, Wheeler E, Glazer NL, Bouatia-Naji N, Gloyn AL (2010). New genetic loci implicated in fasting glucose homeostasis and their impact on type 2 diabetes risk. Nat Genet.

[b8] Gannon M, Herrera PL, Wright CV (2000). Mosaic Cre-mediated recombination in pancreas using the pdx-1 enhancer/promoter. Genesis.

[b9] Georgia S, Bhushan A (2004). Beta cell replication is the primary mechanism for maintaining postnatal beta cell mass. J Clin Invest.

[b10] Georgia S, Hinault C, Kawamori D, Hu J, Meyer J, Kanji M, Bhushan A, Kulkarni RN (2010). Cyclin D2 is essential for the compensatory beta-cell hyperplastic response to insulin resistance in rodents. Diabetes.

[b11] Gu C, Stein GH, Pan N, Goebbels S, Hornberg H, Nave KA, Herrera P, White P, Kaestner KH, Sussel L (2010). Pancreatic beta cells require NeuroD to achieve and maintain functional maturity. Cell Metab.

[b12] Hohmeier HE, Mulder H, Chen GX, Henkel-Rieger R, Prentki M, Newgard CB (2000). Isolation of INS-1-derived cell lines with robust ATP-sensitive K+ channel-dependent and -independent glucose-stimulated insulin secretion. Diabetes.

[b13] Jensen J, Serup P, Karlsen C, Nielsen TF, Madsen OD (1996). mRNA profiling of rat islet tumors reveals nkx 6.1 as a beta-cell-specific homeodomain transcription factor. J Biol Chem.

[b14] Jonsson J, Carlsson L, Edlund T, Edlund H (1994). Insulin-promoter-factor 1 is required for pancreas development in mice. Nature.

[b15] Kang HS, Kim YS, ZeRuth G, Beak JY, Gerrish K, Kilic G, Sosa-Pineda B, Jensen J, Pierreux CE, Lemaigre FP (2009). Transcription factor Glis3, a novel critical player in the regulation of pancreatic beta-cell development and insulin gene expression. Mol Cell Biol.

[b16] Kim YS, Nakanishi G, Lewandoski M, Jetten AM (2003). GLIS3, a novel member of the GLIS subfamily of Kruppel-like zinc finger proteins with repressor and activation functions. Nucleic Acids Res.

[b17] Kushner JA, Ciemerych MA, Sicinska E, Wartschow LM, Teta M, Long SY, Sicinski P, White MF (2005). Cyclins D2 and D1 are essential for postnatal pancreatic beta-cell growth. Mol Cell Biol.

[b18] Liu C, Li H, Qi L, Loos RJ, Qi Q, Lu L, Gan W, Lin X (2011). Variants in GLIS3 and CRY2 are associated with type 2 diabetes and impaired fasting glucose in Chinese Hans. PLoS One.

[b19] Offield MF, Jetton TL, Labosky PA, Ray M, Stein RW, Magnuson MA, Hogan BL, Wright CV (1996). PDX-1 is required for pancreatic outgrowth and differentiation of the rostral duodenum. Development.

[b20] Ohlsson H, Karlsson K, Edlund T (1993). IPF1, a homeodomain-containing transactivator of the insulin gene. EMBO J.

[b21] Olson LK, Redmon JB, Towle HC, Robertson RP (1993). Chronic exposure of HIT cells to high glucose concentrations paradoxically decreases insulin gene transcription and alters binding of insulin gene regulatory protein. J Clin Invest.

[b22] Oster A, Jensen J, Serup P, Galante P, Madsen OD, Larsson LI (1998). Rat endocrine pancreatic development in relation to two homeobox gene products (Pdx-1 and Nkx 6.1). J Histochem Cytochem.

[b23] Pang K, Mukonoweshuro C, Wong GG (1994). Beta cells arise from glucose transporter type 2 (Glut2)-expressing epithelial cells of the developing rat pancreas. Proc Natl Acad Sci USA.

[b24] Poitout V, Robertson RP (2002). Minireview: secondary beta-cell failure in type 2 diabetes – a convergence of glucotoxicity and lipotoxicity. Endocrinology.

[b25] Poitout V, Robertson RP (2008). Glucolipotoxicity: fuel excess and beta-cell dysfunction. Endocr Rev.

[b26] Preitner F, Ibberson M, Franklin I, Binnert C, Pende M, Gjinovci A, Hansotia T, Drucker DJ, Wollheim C, Burcelin R (2004). Gluco-incretins control insulin secretion at multiple levels as revealed in mice lacking GLP-1 and GIP receptors. J Clin Invest.

[b27] Rees SD, Hydrie MZ, O'Hare JP, Kumar S, Shera AS, Basit A, Barnett AH, Kelly MA (2011). Effects of 16 genetic variants on fasting glucose and type 2 diabetes in South Asians: ADCY5 and GLIS3 variants may predispose to type 2 diabetes. PLoS One.

[b28] Robertson RP, Harmon J, Tran PO, Poitout V (2004). Beta-cell glucose toxicity, lipotoxicity, and chronic oxidative stress in type 2 diabetes. Diabetes.

[b29] Rudnick A, Ling TY, Odagiri H, Rutter WJ, German MS (1994). Pancreatic beta cells express a diverse set of homeobox genes. Proc Natl Acad Sci USA.

[b30] Sachdeva MM, Stoffers DA (2009). Minireview: meeting the demand for insulin: molecular mechanisms of adaptive postnatal beta-cell mass expansion. Mol Endocrinol.

[b31] Senee V, Chelala C, Duchatelet S, Feng D, Blanc H, Cossec JC, Charon C, Nicolino M, Boileau P, Cavener DR (2006). Mutations in GLIS3 are responsible for a rare syndrome with neonatal diabetes mellitus and congenital hypothyroidism. Nat Genet.

[b32] Thorens B, Weir GC, Leahy JL, Lodish HF, Bonner-Weir S (1990). Reduced expression of the liver/beta-cell glucose transporter isoform in glucose-insensitive pancreatic beta cells of diabetic rats. Proc Natl Acad Sci USA.

[b33] Wang S, Jensen JN, Seymour PA, Hsu W, Dor Y, Sander M, Magnuson MA, Serup P, Gu G (2009). Sustained Neurog3 expression in hormone-expressing islet cells is required for endocrine maturation and function. Proc Natl Acad Sci USA.

[b34] Watanabe N, Hiramatsu K, Miyamoto R, Yasuda K, Suzuki N, Oshima N, Kiyonari H, Shiba D, Nishio S, Mochizuki T (2009). A murine model of neonatal diabetes mellitus in Glis3-deficient mice. FEBS Lett.

[b35] Weir GC, Bonner-Weir S (2004). Five stages of evolving beta-cell dysfunction during progression to diabetes. Diabetes.

[b36] Yang Y, Chang BH, Samson SL, Li MV, Chan L (2009). The Kruppel-like zinc finger protein Glis3 directly and indirectly activates insulin gene transcription. Nucleic Acids Res.

[b37] Yang Y, Chang BH, Yechoor V, Chen W, Li L, Tsai MJ, Chan L (2011). The Kruppel-like zinc finger protein GLIS3 transactivates neurogenin 3 for proper fetal pancreatic islet differentiation in mice. Diabetologia.

