# Supplementary material for: Sustained expression of the transcription factor GLIS3 is required for normal beta cell function in adults
Source: EMBO Mol Med. 2012 Nov 29;5(1):92–104. doi: 10.1002/emmm.201201398 (PMC3569656; doi:10.1002/emmm.201201398)
Supplement: Supplementary file 1 [file emmm0005-0092-SD1.pdf]

EMBO
Molecular Medicine

Manuscript EMM-2012-01398

Sustained expression of the transcription factor GLIS3 is required for normal beta cell function in adults

Yisheng Yang, Benny Hung-Junn Chang, and Lawrence Chan

Corresponding author: Lawrence Chan, Baylor College of Medicine

Review timeline:

Submission date:	24 March 2012
Editorial Decision:	08 May 2012
Revision received:	05 September 2012
Editorial Decision:	26 September 2012
Revision received:	26 September 2012
Accepted:	27 September 2012

Transaction Report:

(Note: With the exception of the correction of typographical or spelling errors that could be a source of ambiguity, letters and reports are not edited. The original formatting of letters and referee reports may not be reflected in this compilation.)

1st Editorial Decision 08 May 2012

Thank you for the submission of your manuscript to EMBO Molecular Medicine. We have now heard back from the three referees whom we asked to evaluate your manuscript. As you will see from the reports below, the referees find the topic of your study of potential interest. However, they raise substantial concerns on your work, which should be convincingly addressed in a major revision of the present manuscript.

Although referees 2 and 3 find the study to be of potential interest, they also raise a number of concerns about the conclusiveness of the results and several technical issues. Referee 1 feels that the study is not novel enough to consider revision. While referees 2 and 3 also consider that the study has some limitations, they suggest several ways to improve the overall significance, adding functional data and overall strengthening the results.

While it is clear that publication of the paper cannot be considered at this stage, given these overall evaluations I would be open to the submission of a substantially revised manuscript. I must stress however, that the referee concerns must be fully addressed and that acceptance of the manuscript would entail a second round of review. I would add that it is particularly important that all of their suggestions are taken on board as we cannot consider its publication otherwise.

Should you decide to revise the manuscript, please be reminded that revised manuscripts should be submitted within three months of a request for revision; they will otherwise be treated as new submissions, except under exceptional circumstances in which a short extension is obtained from the editor.

I look forward to seeing a revised form of your manuscript as soon as possible.

Should you find that the requested revisions are not feasible within the constraints outlined here and choose, therefore, to submit your paper elsewhere, we would welcome a message to this effect.

Yours sincerely,

Editor
EMBO Molecular Medicine

***** Reviewer's comments *****

Referee #1 (Comments on Novelty/Model System):

Globally, this manuscript is not innovative. The fact that Glis 3 is important for insulin gene expression is already known. Data on the role of Glis3 in beta cell proliferation and apoptosis are preliminary and unclear. Finally, this study is not so well performed on a technical point of view.

Referee #1 (Other Remarks):

In this manuscript, the authors are analyzing the function of the transcription factor GLIS3 in adult pancreatic islets. They are using a number of different animal models: GLIS3+/- mice under normal diet or high fat diet; mice deleted in GLIS3 in adult beta cells. In these models, they are analyzing mouse glycemic status, insulin expression, beta cell proliferation and apoptosis and proliferation, among other parameters.
From this study, we observe that insulin gene expression decreases in GLIS3-deficient mice, but this is expected based on previous literature and the role of GLIS3 as an insulin gene transactivator. We also observe that proliferation and cell survival are modified in GLIS3-deficient mice, but this seems to be indirect effects and the mechanism is poorly defined. In conclusion, information described from this manuscript is not innovative. More specific points are described below:

- Fig. 3C: I was surprised to read that Ccnd2 is decreased in Glis3+/- when compared to Glis3+/+ both under control diet, while beta cell proliferation, measured by PCNA staining is similar (Fig. 3B). In my view, this weakens the demonstration that Ccnd2 could be the explanation for some of the observed phenotype.
Fig. 3D: How does Fig. 3D fit with Fig. 7H. This is not fully clear
Fig. 3F: the quality of the figure is extremely poor.
Page 9, end of first paragraph. I understand that finally, Glis3 is neither directly implicated in beta cell apoptosis, nor in beta cell proliferation. If this is the case, Glis3 is only implicated in insulin gene expression, which is already known (see for example: Kang et al, Transcription factor Glis3, a novel critical player in the regulation of pancreatic beta-cell development and insulin gene expression. Mol Cell Biol. 2009 Dec;29(24):6366-79; Yang et al: The Kr¸ppel-like zinc finger protein Glis3 directly and indirectly activates insulin gene transcription. Nucleic Acids Res. 2009 May;37(8):2529-38.)
Page 9, end of the page: In situ hybridization could be tested/used if antibodies are not available.
Fig. 7: What is the vehicle?
Fig. 7G and text page 10: "PCNA+ islet cells tended to be lower in...". It is not different and does not fit with Fig. 3D.
Fig. 7: What is the meaning of the decreased Ngn3 expression as it is mainly a gene expressed during development. Again, this figure demonstrates that Glis3 is important for insulin gene expression, which is quite well known. In my view, data supporting other roles are weak.

Additional comments:
- Fig.1C: glycemia at time zero in control mice seems very high. Is it expected?
- Page 5 and related figures: beta cell mass should be in grams, not in %. It should be reported to

pancreatic weight that needs to be measured. The way beta cell mass was quantified should be described in the methods. A reference is not sufficient.
- Fig. 2: how to explain that in control mice, high fat diet increases insulin content by less than two fold, while insulin mRNA is increased by five fold.
Fig. 5: the quality of PDX1 and NKX6.1 staining is very poor
Results that relate to cleaved caspase 3: the quality of the staining is not convincing. Additional markers are needed to support the claims. The strong background in the control groups surprised me. What was expected for the control groups?
Fig. 5B: What is the phenotype of the cells in Fig. 5B and 5D. They look negative for all tested markers. Are they dead cells?
Discussion on page 13, end: Type 1 diabetes does not really involve a defect in beta cell function as beta cells are destroyed.

Referee #2 (Comments on Novelty/Model System):

The models used in this manuscript were adequate to address the questions at hand. I feel that this is an overall interesting manuscript, and if revised appropriately, would be suitable for publication. Some of my concerns arise from the incompleteness of the characterizations performed by the authors, and I have made several recommendations that should be feasible. Most notably, the regulation of ccnd2 by Glis3 needs more evaluation.

Referee #2 (Other Remarks):

In this interesting manuscript by Yang, et al., the authors describe the effects of Glis3 heterozygosity on the response to high fat diet feeding in mice, and the effect of cell-specific deletion of Glis3 on metabolic regulation. The authors show a heretofore unappreciated role for Glis3 in islet proliferation in response to high fat diet feeding, and a role for Glis3 in maintenance of normal islet function in the adult. Overall, the studies provide important new information relevant to islet biology and metabolism, and appear to be carefully performed. However, there are some issues that require further attention and additional experimentation:

1. The response to HFD feeding is interesting, and lack of cell mass accrual is quite striking. The assignment of this phenotype to the rather modest reduction in ccnd2 requires a bit more clarification. First, as the authors are aware, many of the cell cycle regulators are typically themselves regulated at the level of mRNA translation, and the mRNA levels themselves may not always be reflective of the protein levels. Thus, a more complete characterization will require assessment of key cell cycle regulators at the protein level (either immunoblot of islet extracts or immunofluorescence). In this case, at minimum the authors should show protein levels of Cyclin D2, and perhaps consider also showing levels of other cdks and cyclins, and possibly also consider showing p16 and p18 levels (considering a recent report by Alonzo and colleagues that saturated fatty acids inhibit cell replication via p16/p18).

2. The suggestion that Glis3 controls transcription of ccnd2 is circumstantial. Whereas the phenotype of the heterozygous mice is suggestive, and the EMSA and ChIP assays show occupancy of the promoter, what is missing are functional data to back up this contention. It should be relatively straightforward to perform complementary assays in cell lines to show that ectopic expression of Glis3 activates ccnd2 using either real-time PCR or a luciferase reporter. A detailed promoter "bashing" experiment is not necessary, but some level of functional data would be helpful.

3. In the cell knockout mice, I appreciate the authors' recognition that the increased apoptotic rate is inconsistent with the data obtained from the HFD mice, and that hyperglycemia may account for this. Nevertheless, considering that the HFD Glis3+/- mice were also hyperglycemic, why wasn't an increased apoptotic rate observed in these mice? One possibility is that TUNEL and cleaved caspase 3 immunostaining is not very sensitive or is likely to miss the temporal state of many dying cells. Again here, a complementation approach in cell lines may provide some insight (e.g. silencing of Glis3 in INS-1 cells might show a reduction in proliferation but no change in apoptosis).

4. Notwithstanding the lack of change in PCNA staining in the cell knockouts, it would be

important for the authors to show whether ccnd2 expression is altered in these mice, and also whether ccnd2 protein is altered. Again, it is possible that a reduction in proliferation, combined with the increase in glucose-induced apoptosis, contributes to the loss of cells in this model.

Minor:
5. The authors should justify why TUNEL staining is used in some cases (e.g. Fig. 7), and cleaved caspase 3 staining in others (e.g. Fig. 6). One concern that arises is simply that the two techniques may show different sensitivity, and as such, cross comparisons may or may not be valid.

6. The EMSA in Fig. 3F requires some clarification that may be addressed with better controls. For example, is the large band at the bottom of the gel the free probe? If so, why is this not evident in the first lane and with the -160 probe? It would be important to show lanes in which control reticulocyte lysate (i.e. lysate with a control protein such as luciferase or some other transcription factor) is added to each probe. This would also allow the unambiguous identification of the presumed shifted complex as containing Glis3. Also, expression of Glis3 in the lysate should be shown (by radiolabeling) or at least it should be mentioned that Glis3 protein production was verified in the lysate.

Referee #3 (Comments on Novelty/Model System):

Commendable use of diet-treated heterozygous mice as well as inducible beta cell-specific ablation of the diabetes risk gene under study

Referee #3 (Other Remarks):

This paper describes the consequences of global haplo-insufficiency or inducible beta cell-specific ablation of the transcription factor Gis3 in the mouse. GWAS have identified Gis3 as a susceptibility gene in type 2 diabetes. The gene has also been linked to sporadic type 1 diabetes. The authors have previously shown that the Gis3 k.o. mouse dies in the neonatal period from acute diabetes. Now they report on the development of diabetes after high fat feeding of Glis3+/ mice and on the rapid appearance of severe insulin deficiency after injection of tamoxifen in Glis3fl/fl/Pdx1CreERT+ mice. Ten days after tamoxifen, there was already suppression of insulin expression and diabetes, which was aggravated further after 8 weeks. At the latter time, there was hardly any insulin remaining in the islets (immunostaining) and beta cell apoptosis occurred. In the heterozygous mice, beta cell replication and hypertrophy in response to the fat-induced insulin resistance was markedly impaired. The latter was claimed to be due to deficient activation of the Gis3 target cyclin D2 and that Glis3 binds to specific response elements in the cyclin D2 promoter (EMSA and CHIP analyses). Ten days after tamixofen injection in the inducible model, there was suppression of selected beta cell genes, in particular insulin while plasma insulin and blood glucose were normal. Finally, Gis3 was shown to bind in vivo to the insulin promoter. The paper is topical and the experiments well conducted. The animal models are convincingly phenotyped and the results advance our understanding of the beta cell actions of this diabetes risk gene.

Specific comments
1. Fig.3C: While Gis3 heterozygous mice had lower islet cyclin D2 mRNA, cyclin D3 was increased. The explanation for the decreased beta cell proliferation after high fat feeding remains obscure and this should be discussed.
2. It is a pity that the authors did not measure insulin secretion in the islets of the haplo-insufficient Gis3. This is necessary to establish whether only the reduction of insulin expression and biosynthesis or other factors as well determine the very low plasma insulin levels after high fat feeding.
3. Fig. 7A, B, E: Ten days after injection of tamoxifen, insulin mRNA and pancreatic insulin staining were markedly reduced without any changes in plasma glucose and insulin. These results must be substantiated by measurements of pancreatic insulin content and/or islet insulin content. This is necessary to understand why the marked loss of pancreatic insulin does not result in changes in glucose homeostasis. Why did the authors not perform glucose tolerance tests on these animals?
4. There is an obvious lack of information regarding insulin secretion from isolate islets. The authors did isolate islets 10 days after tamoxifen injection. At this time, some key beta cell genes were

suppressed while Pdx1, MAFA etc were not affected. For a better understanding of the development of diabetes, it would be important to measure glucose-stimulated insulin secretion in islets at this early stage to allow distinction between simple insulin deficiency or defective insulin secretion.

1st Revision - authors' response 05 September 2012

We appreciate the referees' and editors' constructive comments and suggestions on our manuscript entitled "Sustained expression of the transcription factor GLIS3 is required for normal beta cell function in adults" (EMM-2012-01398). We have performed many additional experiments in response to these comments and have revised the manuscript accordingly. We appreciate the opportunity to revise our work and believe that we have addressed all comments and made appropriate revisions. Below is a point-by-point response to the reviewers' and editors' comments.

Point-by-point response to reviewers' comments:

Referee #1 (Comments on Novelty/Model System):

Globally, this manuscript is not innovative. The fact that Glis 3 is important for insulin gene expression is already known. Data on the role of Glis3 in beta cell proliferation and apoptosis are preliminary and unclear. Finally, this study is not so well performed on a technical point of view.

Response: We apologize that our writing was not explicit enough such that referee# 1 may have missed the significance of the work. After the Glis3 gene was first reported to be a locus for neonatal diabetes about six years ago, many GWAS studies have since found the locus to be strongly associated with type 1 and type 2 diabetes in adult populations. Diabetes in adults encompasses multiple etiological factors. In this manuscript, we have dissected the multi-faceted action of Glis3 in the adult pancreas, and uncovered specific defects in Glis3 haploinsufficiency that may underlie type 2 diabetes. We note that referee #2 found our work to be "an overall interesting manuscript" and referee 3# opined that "the animal models are convincingly phenotyped and the results advance our understanding of the beta cell actions of this diabetes risk gene".

Referee #1 (Other Remarks):

In this manuscript, the authors are analyzing the function of the transcription factor GLIS3 in adult pancreatic islets. They are using a number of different animal models: GLIS3+/- mice under normal diet or high fat diet; mice deleted in GLIS3 in adult beta cells. In these models, they are analyzing mouse glycemic status, insulin expression, beta cell proliferation and apoptosis and proliferation, among other parameters.

From this study, we observe that insulin gene expression decreases in GLIS3-deficient mice, but this is expected based on previous literature and the role of GLIS3 as an insulin gene transactivator. We also observe that proliferation and cell survival are modified in GLIS3-deficient mice, but this seems to be indirect effects and the mechanism is poorly defined. In conclusion, information described from this manuscript is not innovative. More specific points are described below:

- Fig. 3C: I was surprised to read that Ccnd2 is decreased in Glis3+/- when compared to Glis3+/+ both under control diet, while beta cell proliferation, measured by PCNA staining is similar (Fig. 3B). In my view, this weakens the demonstration that Ccnd2 could be the explanation for some of the observed phenotype.

Response: In response to the referees' comments, we have performed many additional experiments (presented in new Fig. 3, and Supplementary Fig. 7) that fully support our finding on the role of Ccnd2. Not only did we repeat and confirm our previous observations, we further found that Glis3 knockdown in INS-1 derived 832/13 cells led to a significant reduction in the expression of Ccnd2 both at the mRNA and the protein level (see Fig. 3C and H); beta cell proliferation, reflected by BrdU+ cells, remained unchanged (Supplementary Fig. 7). Data from the multi-pronged experiments allowed us to conclude that sustained Glis3 and Ccnd2 expression is required for HFD-induced beta cell proliferation, but not for beta cell proliferation under basal conditions.

Fig. 3D: How does Fig. 3D fit with Fig. 7H. This is not fully clear

Response: We showed that both Ccnd2 mRNA (new Fig. 3B) and protein (Fig. 3G) were down regulated in beta cell specific Glis3 deficient mice. As indicated in the response to the comment above, we confirmed these findings in Glis3 knockdown INS-1 derived 832/13 cells.

Fig. 3F: the quality of the figure is extremely poor.

Response: We have enhanced the quality of the EMSA (new Fig. 3J)

Page 9, end of first paragraph. I understand that finally, Glis3 is neither directly implicated in beta cell apoptosis, nor in beta cell proliferation. If this is the case, Glis3 is only implicated in insulin gene expression, which is already known (see for example: Kang et al, Transcription factor Glis3, a novel critical player in the regulation of pancreatic beta-cell development and insulin gene expression. Mol Cell Biol. 2009 Dec;29(24):6366-79; Yang et al: The Krüppel-like zinc finger protein Glis3 directly and indirectly activates insulin gene transcription. Nucleic Acids Res. 2009 May;37(8):2529-38.)

Response: In the current study, we showed evidence that Glis3 is not required for beta cell survival or basal beta cell proliferation in beta cell-specific Glis3 knockout mice (new Fig. 7E and G) or in Glis3 knockdown INS-1 derived 832/13 cells (Fig. 7F and Supplementary Fig. 7). On the other hand, sustained Glis3 expression is required for normal insulin gene transcription (Fig. 4-7) and HFD-induced beta cell proliferation and mass expansion (Fig. 1 and 2).

Page 9, end of the page: In situ hybridization could be tested/used if antibodies are not available.

Response: We have verified the Pdx1-CreERT deletion efficiency by both q-RT-PCR in isolated islets (new Fig.7A) and *in situ* hybridization in pancreas sections (Supplementary Fig. 5).

Fig. 7: What is the vehicle?

Response: The vehicle was peanut oil with 10% ethanol used to dissolve tamoxifen, which was described in MATERIALS AND METHODS and in figure legend.

Fig. 7G and text page 10: "PCNA+ islet cells tended to be lower in...". It is not different and does not fit with Fig. 3D.

Response: We confirmed this finding in Glis3 knockdown INS-1 derived 832/13 cells using BrdU labeling (Supplementary Fig. 7). In our view, sustained Glis3 and Ccnd2 expression is required for HFD-induced beta cell proliferation, but not for beta cell proliferation under basal conditions.

Fig. 7: What is the meaning of the decreased Ngn3 expression as it is mainly a gene expressed during development. Again, this figure demonstrates that Glis3 is important for insulin gene expression, which is quite well known. In my view, data supporting other roles are weak.

Response: The reduction of *Ngn3* mRNA in the adult islets of beta cell-specific *Glis3* deficient mice (new Fig.7A) suggests that *Ngn3* may have contributed to *Glis3*'s effects on adult beta cell function. We note, however, that loss of *Ngn3* in adult mice was reported to cause relatively subtle effects on beta cell function in adult mice (*Wang S, et al. Proc Natl Acad Sci U S A. 2009;106(24):9715-20.*) Thus, given the very robust direct transactivation of *Glis3* on the insulin gene in the adult pancreas, these results indicate that *Ngn3* is unlikely to play a major role in the precipitous loss of insulin production and the resultant fulminant diabetes that occurs after *Glis3* deletion in the adult beta cells.

Additional comments:
- Fig.1C: glycemia at time zero in control mice seems very high. Is it expected?

Response: It is an expected level as we frequently see this blood glucose level in 5 month old wild-type mice (6 hours fasting).

- Page 5 and related figures: beta cell mass should be in grams, not in %. It should be reported to pancreatic weight that needs to be measured. The way beta cell mass was quantified should be described in the methods. A reference is not sufficient.

Response: We agree with these comments. We have specified the meaning of insulin positive cell area and the method for quantification in the revised text.

- Fig. 2: how to explain that in control mice, high fat diet increases insulin content by less than two fold, while insulin mRNA is increased by five fold.

Response: In the original submission, we measured insulin content in whole pancreas. In this revision, we further measured insulin content in isolated islets and it showed that HFD increased islet insulin content by ~2.5 folds in control mice (see panel F in a much expanded new Fig. 2), which is consistent with the percentage of insulin positive cell area (indicating beta cell mass) (new Fig. 2A and B). The discrepancy between insulin content and insulin mRNA is consistent with fine control at both transcriptional and posttranscriptional levels.

Fig. 5: the quality of PDX1 and NKX6.1 staining is very poor

Response: We have improved the staining of PDX1 and NKX6. The merged images are now shown in new Fig. 5G and H. The individual images are shown in Supplementary Fig. 3.

Results that relate to cleaved caspase 3: the quality of the staining is not convincing. Additional markers are needed to support the claims. The strong background in the control groups surprised me. What was expected for the control groups?

Response: We noted that there was higher background with cleaved caspase 3 staining in the pancreas. We therefore performed TUNEL assays and quantified the positive cells in each model, including TAM 8 weeks (new Fig. 5I), TAM + insulin pellet (Fig.6P-R) and TAM 10 days (Fig. 7E).

Fig. 5B: What is the phenotype of the cells in Fig. 5B and 5D. They look negative for all tested markers. Are they dead cells?

Response: Histological examination of the pancreas revealed a markedly reduced islet density in the TAM-treated *Glis3$^{fl/fl}$/Pdx1Cre^{ERT+}*mice for 8 weeks; the residual islets were small and severely disorganized (new Fig. 5A and B). In new Fig 5B, D, F, H, the different islet cells showed distinct phenotypes consistent with cells in different stages from relatively normal cells to dying and dead cells resulting from insulin deficiency and hyperglycemia-induced glucotoxicity.

Discussion on page 13, end: Type 1 diabetes does not really involve a defect in beta cell function as beta cells are destroyed.

Response: It is well accepted that absolute and relative insulin deficiency underlies type 1 and 2 diabetes.

Referee #2 (Comments on Novelty/Model System):

The models used in this manuscript were adequate to address the questions at hand. I feel that this is an overall interesting manuscript, and if revised appropriately, would be suitable for publication. Some of my concerns arise from the incompleteness of the characterizations performed by the authors, and I have made several recommendations that should be feasible. Most notably, the regulation of ccnd2 by Glis3 needs more evaluation.

Response: We appreciate the referee's comments. We have performed many additional experiments to further evaluate the regulation of Ccnd2 by Glis3 (see individual responses below for details)

Referee #2 (Other Remarks):

In this interesting manuscript by Yang, et al., the authors describe the effects of Glis3 heterozygosity on the response to high fat diet feeding in mice, and the effect of β cell-specific deletion of Glis3 on metabolic regulation. The authors show a heretofore unappreciated role for Glis3 in islet proliferation in response to high fat diet feeding, and a role for Glis3 in maintenance of normal islet function in the adult. Overall, the studies provide important new information relevant to islet biology and metabolism, and appear to be carefully performed. However, there are some issues that require further attention and additional experimentation:

1. The response to HFD feeding is interesting, and lack of beta cell mass accrual is quite striking. The assignment of this phenotype to the rather modest reduction in ccnd2 requires a bit more clarification. First, as the authors are aware, many of the cell cycle regulators are typically themselves regulated at the level of mRNA translation, and the mRNA levels themselves may not always be reflective of the protein levels. Thus, a more complete characterization will require

assessment of key cell cycle regulators at the protein level (either immunoblot of islet extracts or immunofluorescence). In this case, at minimum the authors should show protein levels of Cyclin D2, and perhaps consider also showing levels of other cdks and cyclins, and possibly also consider showing p16 and p18 levels (considering a recent report by Alonzo and colleagues that saturated fatty acids inhibit β cell replication via p16/p18).

Response: We agree with the referee. We have conducted western blotting experiments (presented in new Fig. 3) to examine CCND2 protein level in three different models: (1) in islets isolated from *Glis3*$^{+/+}$ and *Glis3*$^{+/-}$ mice fed with a CD or HFD for 20 weeks (Fig. 3F); (2) in islets of *Glis3*$^{fl/fl}$*/Pdx1Cre*$^{ERT+}$ mice treated with TAM (newly diabetic) or vehicle (new Fig. 3G) (3) in INS-1 derived 832/13 cells transfected with control siRNA or *Glis3* siRNA for 48 h (Fig. 3H). The pattern of change at protein level by blotting is very similar to that observed at the mRNA level by qRT-PCR.

We also examined the mRNA expression of *Cdkn2a (P16)* and *Cdkn2c (P18)* in the islets of *Glis3*$^{+/+}$ and *Glis3*$^{+/-}$ mice fed with a CD or HFD for 20 weeks as suggested (Fig. 3A). We found that the mRNA expression of *Cdkn2a* was up regulated in the islets of *Glis3*$^{+/+}$ HFD and *Glis3*$^{+/-}$ CD mice, compared to *Glis3*$^{+/+}$ CD mice. No difference was observed between *Glis3*$^{+/+}$ HFD and *Glis3*$^{+/-}$ HFD. It seems that *Cdkn2a* does not contribute to the effect of Glis3 on beta cell proliferation in response to HFD feeding.

2. The suggestion that Glis3 controls transcription of ccnd2 is circumstantial. Whereas the phenotype of the heterozygous mice is suggestive, and the EMSA and ChIP assays show occupancy of the promoter, what is missing are functional data to back up this contention. It should be relatively straightforward to perform complementary assays in cell lines to show that ectopic expression of Glis3 activates ccnd2 using either real-time PCR or a luciferase reporter. A detailed promoter "bashing" experiment is not necessary, but some level of functional data would be helpful.

Response: We appreciate the referee's comments and agree with them. We have conducted two functional experiments, the results of which support the conclusion that Glis3 controls transcription of Ccnd2. [1] We knocked down Glis3 gene in INS-1 derived 832/13 cells with siRNA and found that the expression of Ccnd2 was significantly down regulated at the mRNA (new Fig. 3C) and protein level (Fig. 3H). [2] We established an 832/13 cell model stably expressing Glis3 (new Fig. 3D) and found that the mRNA expression of Ccnd2 was significantly up regulated compared to control (Fig. 3E).

3. In the beta cell knockout mice, I appreciate the authors' recognition that the increased apoptotic rate is inconsistent with the data obtained from the HFD mice, and that hyperglycemia may account for this. Nevertheless, considering that the HFD Glis3+/- mice were also hyperglycemic, why wasn't an increased apoptotic rate observed in these mice? One possibility is that TUNEL and cleaved caspase 3 immunostaining is not very sensitive or is likely to miss the temporal state of many dying beta cells. Again here, a complementation approach in cell lines may provide some insight (e.g. silencing of Glis3 in INS-1 cells might show a reduction in proliferation but no change in apoptosis).

Response: We noted that there was higher background with cleaved caspase 3 staining in the pancreas; while TUNEL assay presented higher specificity but low sensitivity in the pancreatic islets. As suggested by the referee, we performed TUNEL assays and BrdU staining in Glis3 knockdown INS-1 derived 832/13 cells. No difference was observed for proliferation (Supplementary Fig. 7) and apoptosis (new Fig. 7F). In our view, sustained Glis3 expression is required for HFD-induced beta cell proliferation, but not for basal beta cell proliferation or beta cell survival.

4. Notwithstanding the lack of change in PCNA staining in the beta cell knockouts, it would be important for the authors to show whether ccnd2 expression is altered in these mice, and also whether ccnd2 protein is altered. Again, it is possible that a reduction in proliferation, combined with the increase in glucose-induced apoptosis, contributes to the loss of beta cells in this model.

Response: We have conducted q-RT-PCR and western blotting in the isolated islets of control and beta cell specific Glis3 knockout mice (TAM treated, newly diabetic, blood glucose just reaching 250 mg/dl)) and found that the expression of Ccnd2 was down regulated both at the mRNA (new Fig. 3B) and protein levels (Fig. 3G). As stated in the response to Question 3, the data are consistent

with sustained Glis3 and Ccnd2 expression being required for HFD-induced beta cell proliferation, but not for basal beta cell proliferation.

Minor:
5. The authors should justify why TUNEL staining is used in some cases (e.g. Fig. 7), and cleaved caspase 3 staining in others (e.g. Fig. 6). One concern that arises is simply that the two techniques may show different sensitivity, and as such, cross comparisons may or may not be valid.

Response: We noted that there was higher background with cleaved caspase 3 staining in the pancreas. We have now also included TUNEL assay and quantified the positive cells in each model such as TAM 8 weeks (new Fig. 5I), TAM + insulin pellet (Fig.6P-R) and TAM 10 days (Fig. 7E).

6. The EMSA in Fig. 3F requires some clarification that may be addressed with better controls. For example, is the large band at the bottom of the gel the free probe? If so, why is this not evident in the first lane and with the -160 probe? It would be important to show lanes in which control reticulocyte lysate (i.e. lysate with a control protein such as luciferase or some other transcription factor) is added to each probe. This would also allow the unambiguous identification of the presumed shifted complex as containing Glis3. Also, expression of Glis3 in the lysate should be shown (by radiolabeling) or at least it should be mentioned that Glis3 protein production was verified in the lysate.

Response: We agree with these comments. We have repeated the EMSA rigorously and the results are shown in new Fig. 3J. The large band at the bottom of the gel was free probe, which was indicated in Fig 3J. The *in vitro*-translated GLIS3 (ZFD) was verified by [^{35}S] labeling autoradiography (Supplementary Fig. 1). We included two negative controls: red fluorescent protein (RFP) and dihydrofolate reductase (DHFR), to each biotin-labeled probe (-3670, -1095, and -160) and observed no binding to these unrelated proteins (Supplementary Fig. 2).

Referee #3 (Comments on Novelty/Model System):

Commendable use of diet-treated heterozygous mice as well as inducible beta cell-specific ablation of the diabetes risk gene under study

Referee #3 (Other Remarks):

This paper describes the consequences of global haplo-insufficiency or inducible beta cell-specific ablation of the transcription factor Gis3 in the mouse. GWAS have identified Gis3 as a susceptibility gene in type 2 diabetes. The gene has also been linked to sporadic type 1 diabetes. The authors have previously shown that the Gis3 k.o. mouse dies in the neonatal period from acute diabetes. Now they report on the development of diabetes after high fat feeding of Glis3+/− mice and on the rapid appearance of severe insulin deficiency after injection of tamoxifen in Glis3fl/fl/Pdx1CreERT+ mice. Ten days after tamoxifen, there was already suppression of insulin expression and diabetes, which was aggravated further after 8 weeks. At the latter time, there was hardly any insulin remaining in the islets (immunostaining) and beta cell apoptosis occurred. In the heterozygous mice, beta cell replication and hypertrophy in response to the fat-induced insulin resistance was marked ly impaired. The latter was claimed to be due to deficient activation of the Gis3 target cyclin D2 and that Glis3 binds to specific response elements in the cyclin D2 promoter (EMSA and CHIP analyses). Ten days after tamixofen injection in the inducible model, there was suppression of selected beta cell genes, in particular insulin while plasma insulin and blood glucose were normal. Finally, Gis3 was shown to bind in vivo to the insulin promoter. The paper is topical and the experiments well conducted. The animal models are convincingly phenotyped and the results advance our understanding of the beta cell actions of this diabetes risk gene.

Specific comments
1. Fig.3C: While Gis3 heterozygous mice had lower islet cyclin D2 mRNA, cyclin D3 was increased. The explanation for the decreased beta cell proliferation after high fat feeding remains obscure and this should be discussed.

Response: It is true that Ccnd2 mRNA was down regulated, while Ccnd3 mRNA tended to be increased in the islets of *Glis3*$^{+/-}$ mice (new Fig. 3A), but Ccnd3 mRNA was not changed in the

islets of beta cell specific Glis3 deficient mice (Fig. 3B). Ccnd2, the dominant D-type cyclin in the mouse pancreas (*Georgia S, et al. J Clin Invest, 2004, 114, 963-968. Kushner JA, et al. Mol Cell Biol. 2005,25:3752-3762.*), was reported to be crucial for beta cell mass expansion (*Georgia S, et al. Diabetes, 2010, 59:987-996*). In our view, Glis3 is required for beta cell proliferation and mass expansion in response to HFD feeding at least partly by regulating Ccnd2 transcription.

2. It is a pity that the authors did not measure insulin secretion in the islets of the haplo-insufficient Gis3. This is necessary to establish whether only the reduction of insulin expression and biosynthesis or other factors as well determine the very low plasma insulin levels after high fat feeding.

Response: We agree with the referee. Glucose-stimulated insulin secretion (GSIS) in isolated islets showed no significant difference of insulin secretion in the islets of $Glis3^{+/-}$ and $Glis3^{+/+}$ mice fed a HFD. We note that the islets of $Glis3^{+/-}$showed a nonsignificant trend towards lower insulin secretion compared to those of $Glis3^{+/+}$mice under chow condition (new Fig. 2G), however, if the GSIS was normalized to islet insulin content (Fig. 2F, a common way of quantifying GSIS, *Preitner F, et al., J Clin Invest, 2004,113, 635-645. Gu C, et al., Cell Metab, 2010,11, 298-310*), the $Glis3^{+/-}$ islets showed no defect of insulin secretion (Fig. 2H).

3. Fig. 7A, B, E: Ten days after injection of tamoxifen, insulin mRNA and pancreatic insulin staining were markedly reduced without any changes in plasma glucose and insulin. These results must be substantiated by measurements of pancreatic insulin content and/or islet insulin content. This is necessary to understand why the marked loss of pancreatic insulin does not result in changes in glucose homeostasis. Why did the authors not perform glucose tolerance tests on these animals?

Response: Glucose tolerance test showed that the mice presented a tendency to glucose intolerance at this early time point (10 days after TAM administration) (Supplementary Fig. 6). In our view, the observed phenotypes (the level of insulin mRNA and immunoreactive insulin was reduced with normal plasma insulin and a tendency to glucose intolerance) were only displayed in a short time window; fulminant diabetes supercede rapidly afterwards in these mice.

4. There is an obvious lack of information regarding insulin secretion from isolate islets. The authors did isolate islets 10 days after tamoxifen injection. At this time, some key beta cell genes were suppressed while Pdx1, MAFA etc were not affected. For a better understanding of the development of diabetes, it would be important to measure glucose-stimulated insulin secretion in islets at this early stage to allow distinction between simple insulin deficiency or defective insulin secretion.

Response: $Glis3^{fl/fl}/Pdx1Cre^{ERT+}$ mice developed diabetes over a window of days after TAM administration. The mRNA change pattern of islet-enriched genes (Glis3, Ins1, Ins2, Glut2, Gck, Pdx1, Nkx6-1, Nkx2-2, Ngn3, Neurod1, Isl1 and MafA) was identical in $Glis3^{fl/fl}/Pdx1Cre^{ERT+}$ mice between TAM-induced newly diabetic group (glucose just reaching 250 mg/dl, data not shown) and 10-day TAM group (Fig. 7A). To minimize experimental variation, we chose to perform GSIS in newly diabetic mice (within a time window of 14-20 days) after TAM treatment. GSIS analysis showed decreased insulin release in isolated islets from newly diabetic $Glis3^{fl/fl}/Pdx1Cre^{ERT+}$ mice, compared to those from vehicle-treated mice (Fig. 7H). It is important to note that, to evaluate insulin secretion in Glis3-deficient mice, one must take into consideration the fact that Glis3 is a potent insulin gene transactivator (*Yang Y, et al., Nucleic Acids Res. 2009,37:2529-2538*) and de novo insulin biosynthesis and insulin content of islets are markedly reduced in Glis3-deficient mice. We, therefore, normalized GSIS to islet insulin protein content (Fig. 7I), a strategy commonly used by other investigators in the field (*Preitner F, et al., J Clin Invest, 2004,113, 635-645. Gu C, et al., Cell Metab, 2010,11, 298-310*), and found no significant difference in GSIS of wild-type islets versus Glis3-deficient islets (Fig. 7J).

2nd Editorial Decision 26 September 2012

Thank you for the submission of your revised manuscript "Sustained expression of the transcription factor GLIS3 is required for normal beta cell function in adults" to EMBO Molecular Medicine. We have now received the reports from the reviewers who were asked to re-review your manuscript.

You will be glad to see that the reviewers are now globally supportive and we can proceed with

official acceptance of your manuscript pending the minor change detailed below:

- The description of all reported data that includes statistical testing must state the name of the statistical test used to generate error bars and P values, the number (n) of independent experiments underlying each data point (not replicate measures of one sample), and the actual P value for each test (not merely 'significant' or 'P < 0.05').

I look forward to seeing a revised version of your manuscript as soon as possible.

Yours sincerely,

Editor
EMBO Molecular Medicine

***** Reviewer's comments *****

Referee #2 (Comments on Novelty/Model System):

I feel the authors did an excellent job in revision. The paper is written better, and I feel there is novelty in this study.

Referee #2 (Other Remarks):

I have no further concerns. The authors have done a nice job in the revision.

Referee #3 (Comments on Novelty/Model System):

The authors have extended the paper by performing a substantial number of new experiments. This has improved the manuscript considerably.

Referee #3 (Other Remarks):

The authors have responded satisfactorily to all questions of this reviewer. The paper is now better written and the interpretation of the results is adequate.

2nd Revision - authors' response 26 September 2012

Thank you for your email. We are happy to know that our manuscript (EMM-2012-01398-V2) will be accepted for publication by EMBO Mol Med.

We have now included information on the name of the statistical test used to generate error bars and P values, the number (n) of independent experiments and the actual P value for each test in the figure legends (highlighted in red). To avoid redundancy/confusion in figures with multiple data points, or some with extremely small P values (and thus too many “0”), we retained the use of “P < XX” for some panels in Fig 1E, Fig 2E, Fig 3A, Fig 3G and Fig 7A.

Thank you!
