# Supplementary material for: Sustained expression of the transcription factor GLIS3 is required for normal beta cell function in adults
Source: EMBO Mol Med. 2012 Nov 29;5(1):92–104. doi: 10.1002/emmm.201201398 (PMC3569656; doi:10.1002/emmm.201201398)
Supplement: Supplementary file 2 [file emmm0005-0092-SD2.pdf]

Supplementary information for:

Sustained expression of the transcription factor GLIS3 is required for normal beta cell function in adults

Yisheng Yang [1], Benny Hung-Junn Chang [2], Lawrence Chan [1, 2, 3].
1. Diabetes and Endocrinology Research Center, Division of Diabetes, Endocrinology and Metabolism, Department of Medicine, Baylor College of Medicine, Houston, Texas, USA
2. Department of Molecular & Cellular Biology, Baylor College of Medicine, Houston, Texas, USA
3. Department of Internal Medicine, St. Luke's Episcopal Hospital, Houston, Texas, USA

**Supplementary information**	**Content**
Supplementary Fig 1:	Denaturing Gel Analysis of ^{35}S Labeled Glis3(ZFD) Product.
Supplementary Fig 2:	EMSA using *in vitro* translated GLIS3-ZFD and control proteins red fluorescent protein (RFP) and dihydrofolate reductase (DHFR) with biotin-labeled probes in mouse *Ccnd2* gene promoter.
Supplementary Fig 3:	Immunostaining PDX1 and NKX6-1 in the pancreas of *Glis3$^{fl/fl}$/ Pdx1Cre^{ERT+}* mice 8 weeks after TAM or vehicle administration.
Supplementary Fig 4:	Immunostaining and quantification of cleaved caspase-3 in the pancreas of *Glis3$^{fl/fl}$/Pdx1Cre^{ERT+}* mice eight weeks after TAM (with or without insulin pellets) or vehicle administration.
Supplementary Fig 5:	The mRNA expression of *Glis3* in the pancreas of *Glis3$^{fl/fl}$/ Pdx1Cre^{ERT+}* mice treated with vehicle or TAM for 10 days was analyzed by *in situ* hybridization.
Supplementary Fig 6:	Gavage GTT (1.5g/kg) was performed in *Glis3$^{fl/fl}$/Pdx1Cre^{ERT+}* mice 10 days after TAM or vehicle treatment.
Supplementary Fig 7:	Representative images and percentage of BrdU^{+} cells in INS-1 derived 832/13 cells transfected with control siRNA or *Glis3* siRNA for 48 h.
Supplementary Table 1:	The sequences of qPCR primers and EMSA probes

Supplementary Fig 1

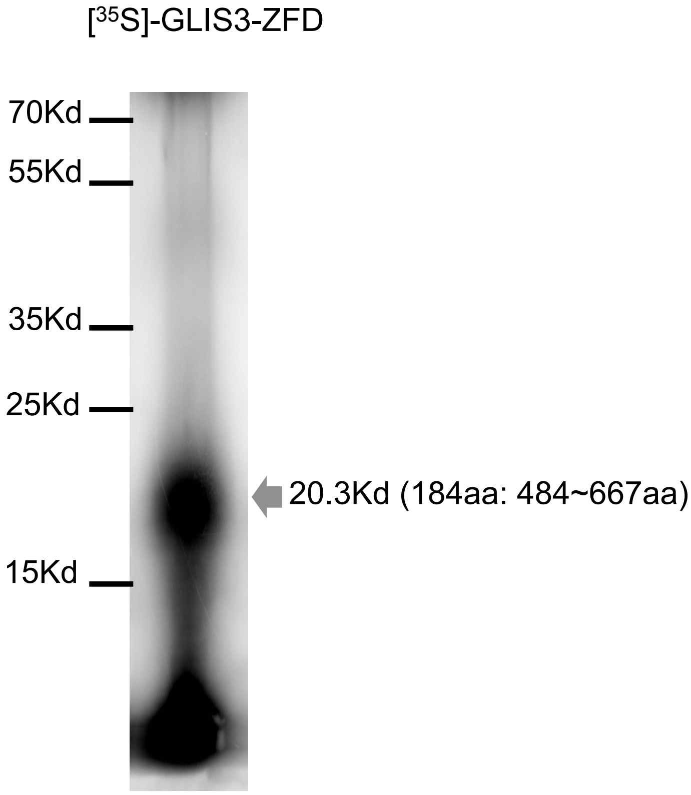

Supplementary Fig 1: Denaturing Gel Analysis of ^{35}S Labeled Glis3(ZFD) Product. [^{35}S]methionine was utilized to label Glis3(ZFD) protein using TNT Quick Coupled Transcription/Translation System. Five microlitter of the denatured sample separated by 10% SDS-PAGE. Expose the gel on Phosphor imager.

Supplementary Fig 2

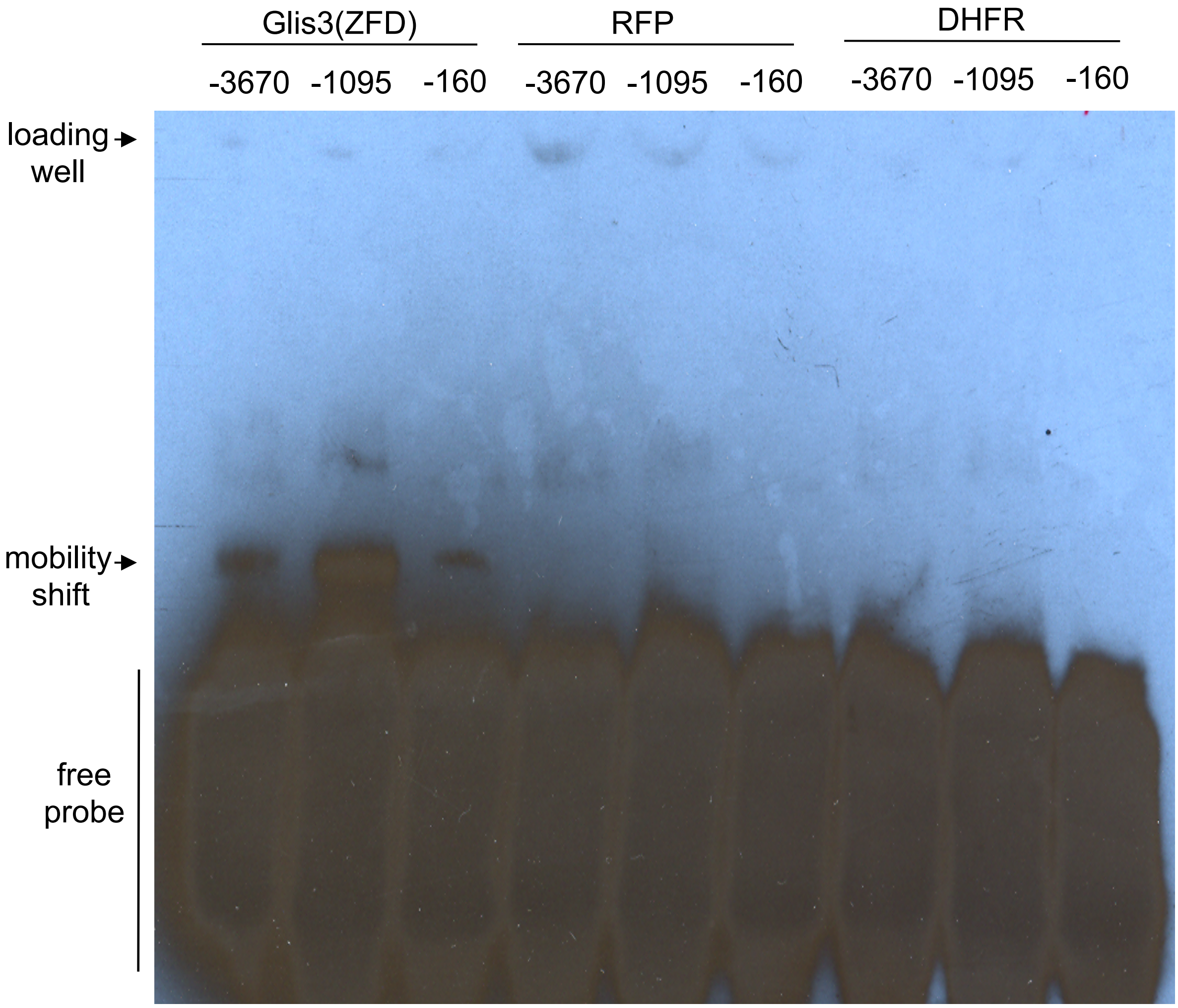

Supplementary Fig 2: EMSA using an *in vitro* translated GLIS3-ZFD peptide was performed with biotin-labeled probes containing putative Glis3REs sequences at -3670, -1095, and -160 in mouse *Ccnd2* gene promoter. *In vitro* translated red fluorescent protein (RFP) and dihydrofolate reductase (DHFR) were used as negative controls.

Supplementary Fig 3

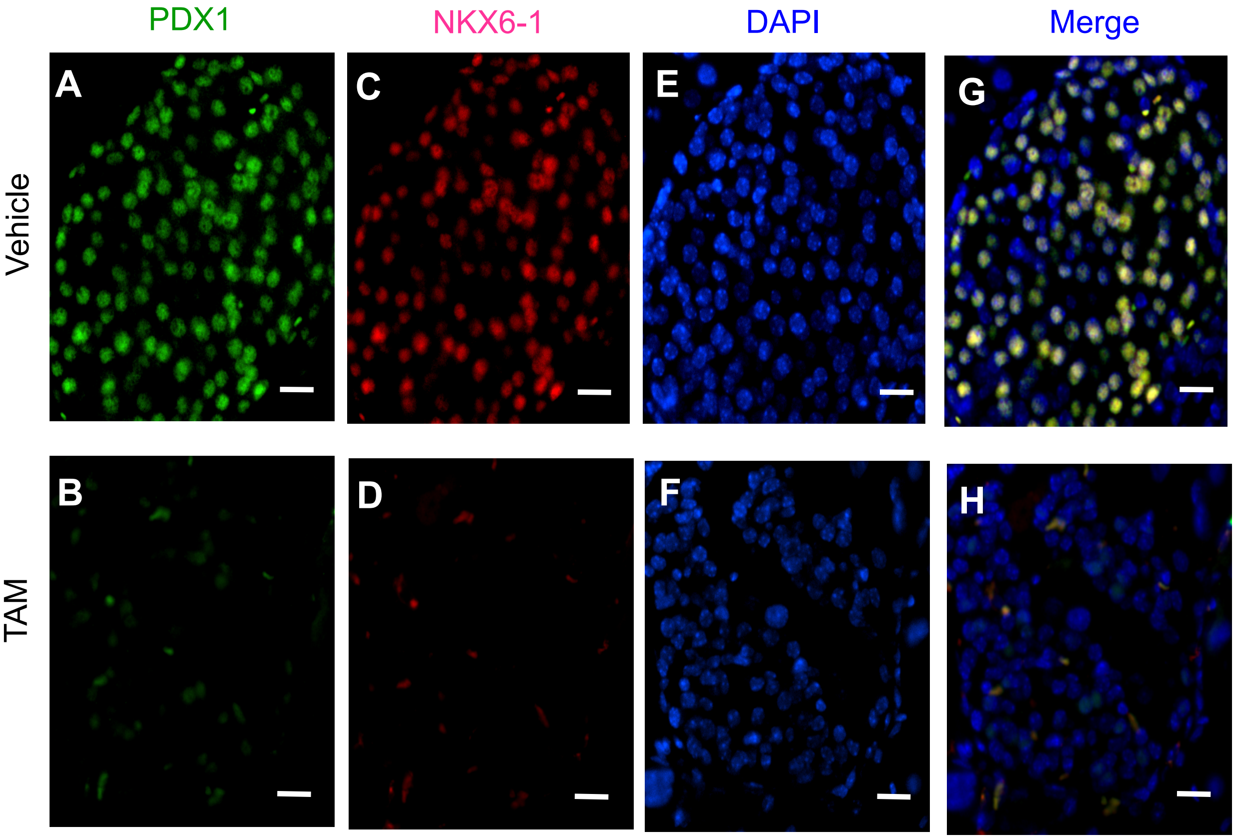

Supplementary Fig 3: Immunostaining PDX1 and NKX6-1 in the pancreas of *Glis3*$^{fl/fl}$/*Pdx1Cre*$^{ERT+}$ mice 8 weeks after TAM or vehicle administration. Scale bar, 25 μm.

Supplementary Fig 4

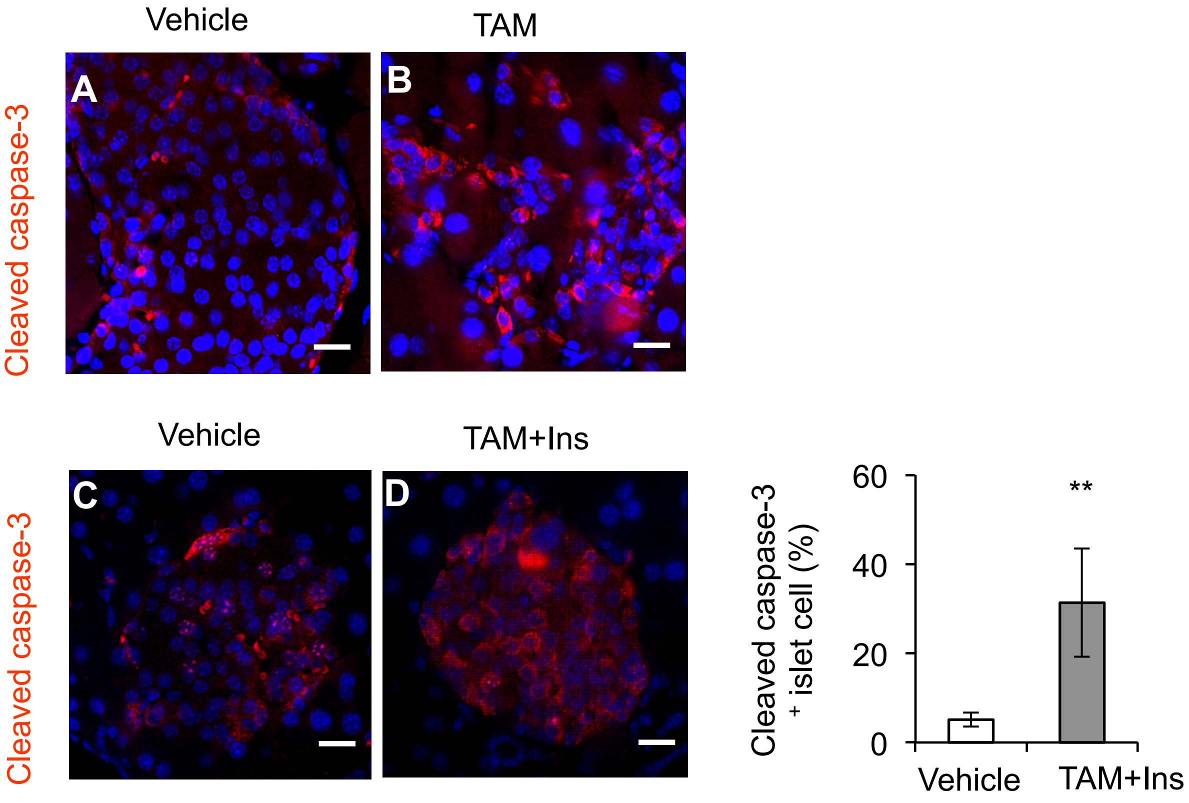

Supplementary Fig 4: (A,B) Immunostaining of cleaved caspase-3 in the pancreas of *Glis3*$^{fl/fl}$/*Pdx1Cre*$^{ERT+}$ mice eight weeks after TAM or vehicle administration. **(C,D)** Immunostaining and quantification of cleaved caspase-3 in the pancreas of *Glis3*$^{fl/fl}$/*Pdx1Cre*$^{ERT+}$ mice eight weeks after vehicle or TAM/ implanted insulin pellets administration (blood glucose < 300 mg/dl). Scale bar, 20 μm. **: P=0.008 versus vehicle-treated mice.

Supplementary Fig 5

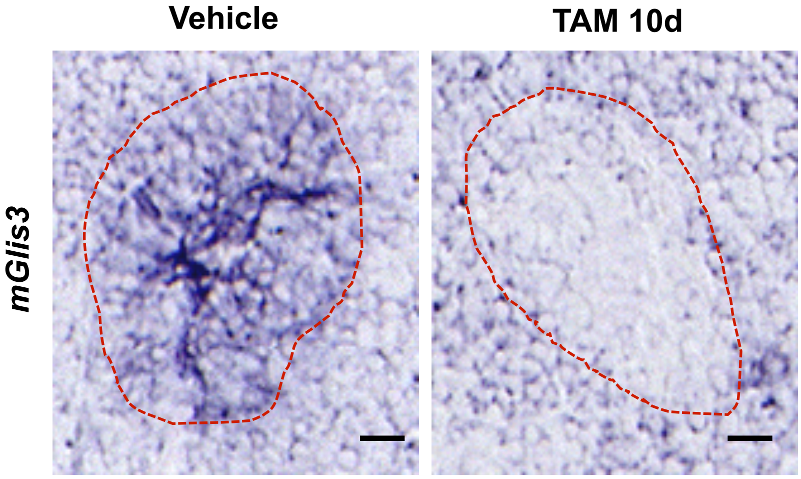

Supplementary Fig 5: The mRNA expression of *Glis3* in the pancreas of *Glis3*$^{fl/fl}$/ *Pdx1Cre*$^{ERT+}$ mice treated with vehicle or TAM for 10 days was analyzed by *in situ* hybridization. Scale bar, 40 μm.

Supplementary Fig 6

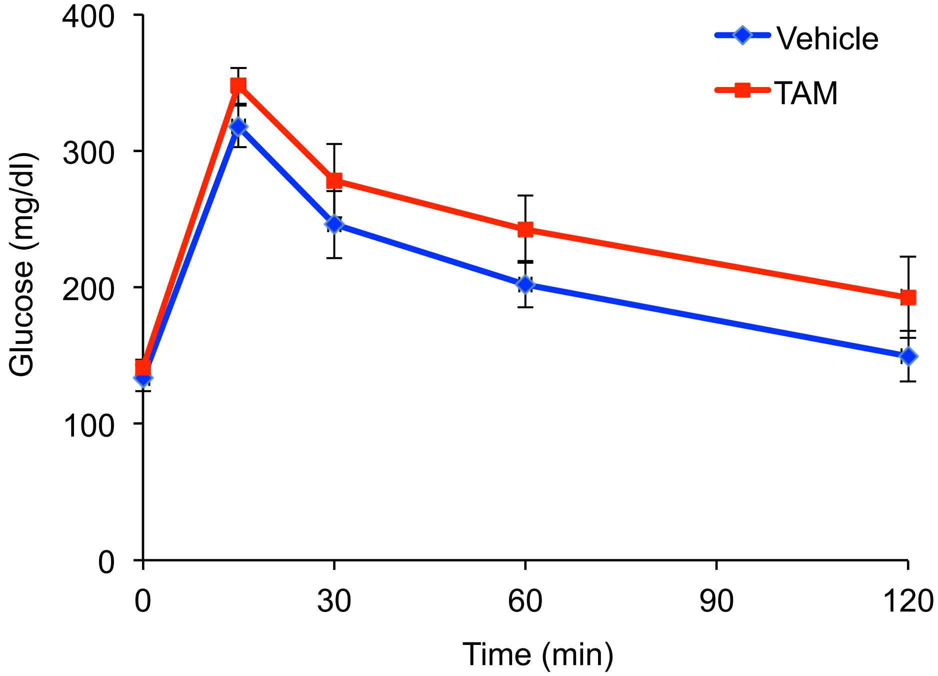

Supplementary Fig 6: Gavage GTT in *Glis3$^{fl/fl}$/Pdx1Cre^{ERT+}* mice 10 days after TAM or vehicle treatment. After 6 h fast, gavage GTT (1.5g/Kg BW) was performed in *these mice*. Plasma glucose was measured at time 0, 15, 30, 60, and 120 min after glucose injection. n=6 for each group.

Supplementary Fig 7

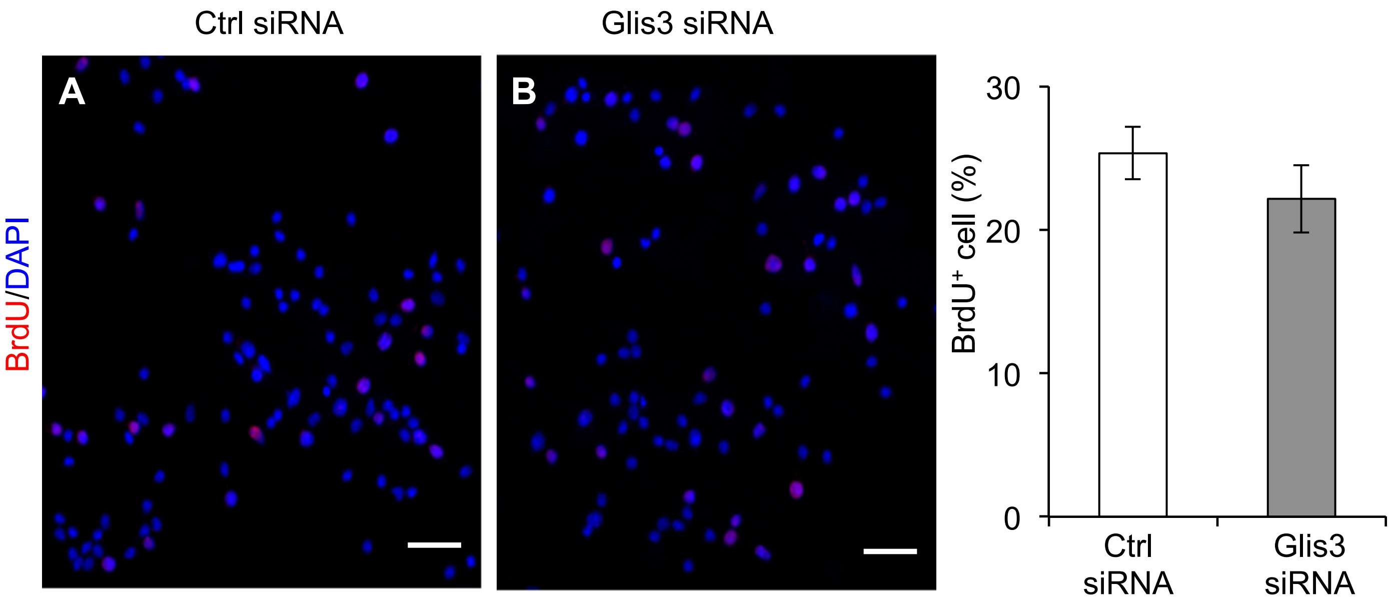

Supplementary Fig 7: Representative images and percentage of BrdU$^+$ cells in INS-1 derived 832/13 cells transfected with control siRNA or *Glis3* siRNA for 48 h. Scale bar, 40 μm.

Supplementary Table 1. The sequences of qPCR primers and EMSA probes

Target	Forward	Reverse
**q-RT-PCR**		
mGlis3	CAGTCCTAGCTTACCGAGG	CTGGGAACTGTGGCTGGATG
mIns1	GGAGCGTGGCTTCTTCTACA	GGTGGGCCTTAGTTGCAGTA
mIns2	TTTGTCAAGCAGCACCTTTG	TCTACAATGCCACGCTTCTG
mPdx1	CTTTCCCGAATGGAACCGAG	GAATTCCTTCTCCAGCTCC
mCcnd1	AGTGCGTGCAGAAGGAGATT	CACAACTTCTCGGCAGTCAA
mCcnd2	GCTGTCCCTGATCCGCAAGC	GGTGATCTTGGCCAGCAGC
mCcnd3	CTGTTGCTGGCCTCCAAGC	TCACTGGGCAGAGACAGGCG
mCdk4	TGGCTGCCACTCGATATGAAC	CCTCAGGTCCTGGTCTATATG
mCdkn1a	TACCCAACTACCAGCTGTGG	ACGGCGCAACTGCTCACTGT
mCdkn1b	CTCTGAGGACCGGCATTTGG	CGTCTGCTCCACAGTGCCAG
mCdkn2a	AGACCCAGGACAGCGAGCTG	GGGTCCTCGCAGTTCGAATC
mCdkn2c	AACGAGTTGGCGTCCGCAGC	AGCACCTCTGAGGAGAAGCC
rCcnd2	CCATGTACCCGCCATCGATG	GGCTGTTAAGCAGCACAGC
mGlut2	GAGATTGGGCCAGGTCCAATC	AACAGGGTGAAGACCAGGAC
mGck	AGCTGGTACGACTTGTGCTG	ACGGCGCACAATGTCGCAGT
mNkx6-1	CTATTCTCTGGGGATGACGG	TCTCGTCGTCAGAGTTCGGGTC
mNkx2-2	TCTACGACAGCAGCGACAAC	TTCCGCTTCTTGCCTGCG
mNeurog3	GTCGGGAGAACTAGGATGGC	GGAGCAGTCCCTAGGTATG
mNeuroD1	GGCTCCAGGGTTATGAGATC	GCATTCATGGCTTCAAGC
mIsl1	CAGATGCGCTCATGAAGGAG	AGCAGCCACCATGGGAGTTC
mMafA	AGCAAGGAGGAGGTCATC	CGTATTTCTCCTTGTACAGG
rmCyclophilin A	CTGTTTGCAGACAAAGTTCCA	AGGATGAAGTTCTCATCCTCA

Supplementary Table 1. The sequences of qPCR primers and EMSA probes (*Continued*)

**ChIP-q-PCR**		
mCcnd2 (-9926)	AACCTCACAGGCAGATGAGG	GCCAGCAAGATACCTGTTCC
mCcnd2 (-3670)	GGAGATGCGAGGTGGGTGAG	AGGAGCCACCCGACAGCAGA
mCcnd2 (-1095)	CATGCTATCTGCACGTGCC	GCATCCTGTGGTCAAGGCTG
mCcnd2 (-160)	GTCAGGCCAGCTGCTGTGCT	CGCTCAGGCAGTGACGCAAG
MIP(-6000)	GTAAGAACACAGGGAAGGCC	CTAATTGGAGCCTAAGGCAC
MIP(-266)	GGACAAAGAAAGCATCACCC	AGCGGATCACTTAGGGCTGG
**EMSA probe**		
mCcnd2 (-3670)	TTCCCAGCTTATCCTCTGCCACGAGGGCTCTACCT	
mCcnd2 (-1095)	ACGCCAACGTGTCCCCCTCTACCAACAGCCTTGAC	
mCcnd2 (-160)	GTTTGGTCAGGCCAGCTGCTGTGCTCCTTAATAAC	
